# Co‐Selection of Low Cadmium Accumulation and High Yield During Tomato Improvement

**DOI:** 10.1002/advs.202505138

**Published:** 2025-07-26

**Authors:** Xingyu Zhang, Mei Qing, Haobo Xu, Jinbao Tao, Fangman Li, Pingfei Ge, Yang Yang, Wenqian Wang, Yongen Lu, Donald Grierson, Zhibiao Ye, Yuyang Zhang

**Affiliations:** ^1^ National Key Laboratory for Germplasm Innovation and Utilization of Horticultural Crops Huazhong Agricultural University Wuhan 430070 China; ^2^ Hubei Hongshan Laboratory Wuhan 430070 China; ^3^ Hubei Key Laboratory of Metabolic Abnormalities and Vascular Aging Wuhan 430022 China; ^4^ Plant Sciences Division School of Biosciences University of Nottingham Sutton Bonington Campus Loughborough LE12 5RD UK

**Keywords:** cadmium, co‐selection, gene identification, genetic hitchhiking, tomato

## Abstract

Enhancing crop production and yield is necessary to feed an increasing population, but cadmium (Cd) accumulation in crops poses a serious threat to human health. It is found that a trend during domestication is for the co‐selection of improved tomato yield and reduced Cd accumulation. A genome‐wide association study (GWAS) of 506 tomato accessions identifies a natural allele, *LCT1^AA^*, which confers low Cd accumulation in the shoots and fruits of tomato. The linkage disequilibrium (LD) analysis reveals a tight linkage between *LCT1^A^* and a large fruit allele *fw3.2^T^*. Evolution analysis shows that *fw3.2* and *LCT1* experience similar selection pressure. Grafting experiments using tomato hypocotyls as the junction point further demonstrate that *LCT1* reduces Cd accumulation primarily through regulation in the root rather than the shoot of the plant. These findings collectively highlight the importance of *LCT1* in regulating Cd accumulation and indicate that the widespread presence of low Cd accumulating types in cultivated tomato is due to genetic hitchhiking and co‐selection of *LCT1^A^* with *fw3.2^T^* during yield breeding

## Graphical abstract

This study finds that large‐fruited tomatoes accumulate less cadmium (Cd) compared to wild tomatoes and cherry tomatoes. This is because, during the improvement process of tomato, the low‐Cd allele *LCT1^A^* and the large fruit allele *fw3.2^T^* are closely linked, leading to a co‐selection event.

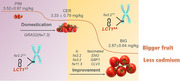

## 1 Introduction

Cadmium (Cd) is a vital industrial metal. However, unregulated wastewater discharges from mining and metal smelting have led to significant environmental releases of Cd, threatening both plant growth and human health.^[^ ^1^, ^2^, ^3^ ^]^ High concentrations of Cd inhibit root development in plants and disrupt their absorption of key nutrients such as iron (Fe), magnesium (Mg), and zinc (Zn).^[^ ^4^, ^5^ ^]^ This results in symptoms including leaf chlorosis, stunted growth, and, in severe cases, plant death.^[^ ^6^, ^7^ ^]^ Even at lower concentrations, Cd can compromise the nutritional quality and safety of agricultural products, posing insidious risks to human health.^[^ ^8^, ^9^ ^]^ Cd primarily enters the human body through the food chain, accumulating predominantly in the kidneys and bones.^[^ ^10^ ^]^ Prolonged exposure can lead to tubular dysfunction, chronic kidney failure, and skeletal issues such as osteoporosis, atrophy, deformities, and bone pain—similar to the “Itai‐itai disease” observed in Japan.^[^ ^11^, ^12^ ^]^ Acute Cd poisoning may present with symptoms such as vomiting, diarrhea, and renal failure, which can be life‐threatening if not treated promptly.^[^ ^13^ ^]^

Plants mitigate Cd toxicity through multiple molecular mechanisms. Transporters in the root cell plasma membrane, like ABC and NRAMP family members, mediate Cd uptake and efflux.^[^ ^14^, ^15^ ^]^ The cell wall, rich in pectin and cellulose, immobilizes Cd, limiting its entry into the cytoplasm.^[^ ^16^ ^]^ Vacuolar membrane transporters, such as the HMA and ABC families, sequester Cd into vacuoles, reducing its impact on cellular metabolism.^[^ ^17^, ^18^ ^]^ Phytochelatins, glutathione (GSH), and flavonoids also form stable complexes with Cd, lowering its toxicity and mobility.^[^ ^19^, ^20^, ^21^ ^]^ Additionally, auxin signaling promotes the root secretion of phytochelatins and organic acids, thereby decreasing heavy‐metal uptake by the roots.^[^ ^22^, ^23^, ^24^ ^]^ These mechanisms work together to construct a defense system against Cd toxicity, involving absorption, transport, immobilization, sequestration, and detoxification.

Tomato (*Solanum lycopersicum*) is the most widely consumed vegetable crop globally.^[^ ^25^ ^]^ The Cd level in consumed tomatoes is deeply intertwined with human health.^[^ ^9^, ^26^ ^]^ However, the genetic mechanism underlying Cd accumulation in tomato is poorly understood. Here, we investigated the Cd accumulation capability of 506 tomato accessions and pinpointed the pivotal gene *LCT1* (*Low Cadmium in Tomato 1*), which encodes flavonoid 3’‐hydroxylase (F3’H), through GWAS. A specific single‐nucleotide polymorphism (SNP3‐A) located in the 3’UTR of the *LCT1* gene was found to regulate its expression, resulting in decreased Cd accumulation in both the shoots and fruits of tomato. Intriguingly, a co‐selection between the low‐Cd allele *LCT1^A^* and the large fruit allele *fw3.2^T^* was observed, potentially contributing to the gradual reduction in Cd accumulation during the domestication and improvement of tomato.

## 2 Results

### 2.1 Cultivated Tomato Varieties Accumulate Less Cd in Shoot Than Wild Ancestors

**Figure 1:**
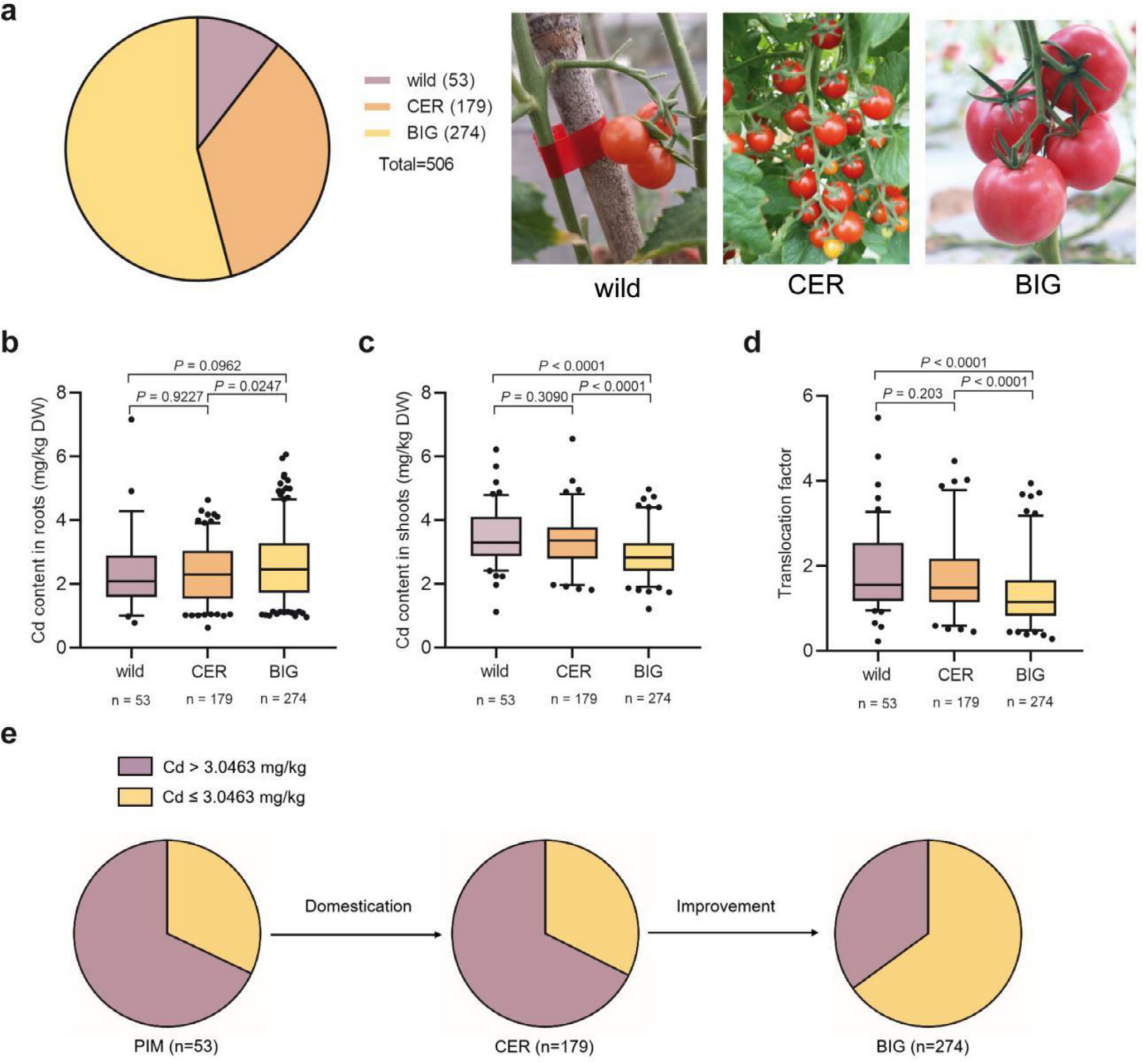
The Cd accumulation and translocation factor in cultivated tomato and its wild relatives. a) Distribution of 506 accessions, including 53 wild accessions (46 *S. pimpinellifolium*, 3 *S. cheesmaniae*, 1 *S. corneliomulleri*, 1 *S. galapagense*, 1 *S. habrochaites*, and 1 *S. neorickii*), 179 CER accessions (*Solanum lycopersicum* var. *cerasiforme*), and 274 BIG accessions (*Solanum lycopersicum*). b,c) The Cd contents in roots (b) and shoots (c) of three groups of 506 accessions after treatment with 1 mg kg^−1^ CdCl_2_ for 30 d. d) The Cd translocation factor of three groups of 506 accessions after treatment with 1 mg kg^−1^ CdCl_2_ for 30 d. e) The frequency distribution of Cd content in wild, CER, and BIG accessions. The median Cd content for the entire tomato population was 3.0463 mg kg^−1^. *n* indicates the number of accessions. In (b–d), the box indicates the range of the percentiles of the total data determined using Tukey's method, the central line indicates the median, the edges of the box represent the first and third quartiles, the whiskers extend to span a 1.5 interquartile range from the edges, and the outer dots are outliers. *n* indicates the number of accessions belonging to each group. *P‐*values were determined by Student's *t*‐test, with *p* < 0.05 indicating a significant difference.

Here, we investigated the differences in Cd accumulation capacity among 506 tomato accessions. Earlier in this study, we observed a high correlation of Cd accumulation between tomato shoots and fruits (Figure S1, Supporting Information). To avoid the potential risk of Cd spreading and contamination, we conducted the Cd treatment in 506 tomato accessions at the seedling stage within a more controlled environment (Figure S2a–c and Table S1, Supporting Information). These 506 accessions represent various geographical origins and genetic backgrounds, consisting of 53 wild accessions, 179 domesticated accessions of *Solanum lycopersicum var. cerasiforme* (CER), and 274 improved accessions of *Solanum lycopersicum* (BIG)^[^ ^27^, ^28^ ^]^ (**Figure** **1**a). We found that the Cd content in the shoots and the Cd translocation factor for the BIG group were significantly lower than those of the wild and CER groups. No significant differences were observed in the Cd contents of the roots and shoots, or in the translocation factor between the wild and CER groups (Figure 1b–d; Table S2, Supporting Information).

Taking the median value (3.0463 mg kg^−1^) of Cd content in the shoots of 506 tomato accessions as the threshold, we found that the proportion of “low Cd” tomato (Cd content < 3.0463 mg kg^−1^) increased gradually from the wild group to the CER group and then to the BIG group, especially during the transition from CER to BIG, where it increased from 32.40% to 64.96% (Figure 1e). This discovery suggests that, unintentionally, as humans have domesticated and improved tomato yields, they have preserved the excellent trait of “low Cd” during the process. This is promising as it indicates that we can use existing cultivars for breeding to rapidly develop low Cd accumulating cultivars. Thus, the domestication and improvement of species for higher yields have not always resulted in the loss of desirable but invisible traits such as flavor^[^ ^29^ ^]^ or stress resistance^[^ ^30^, ^31^ ^]^ and may, fortunately, have led to the preservation of outstanding traits like “low Cd”.

### 2.2 Identification of a Major Locus for Low Cd Accumulation in Tomato

**Figure 2:**
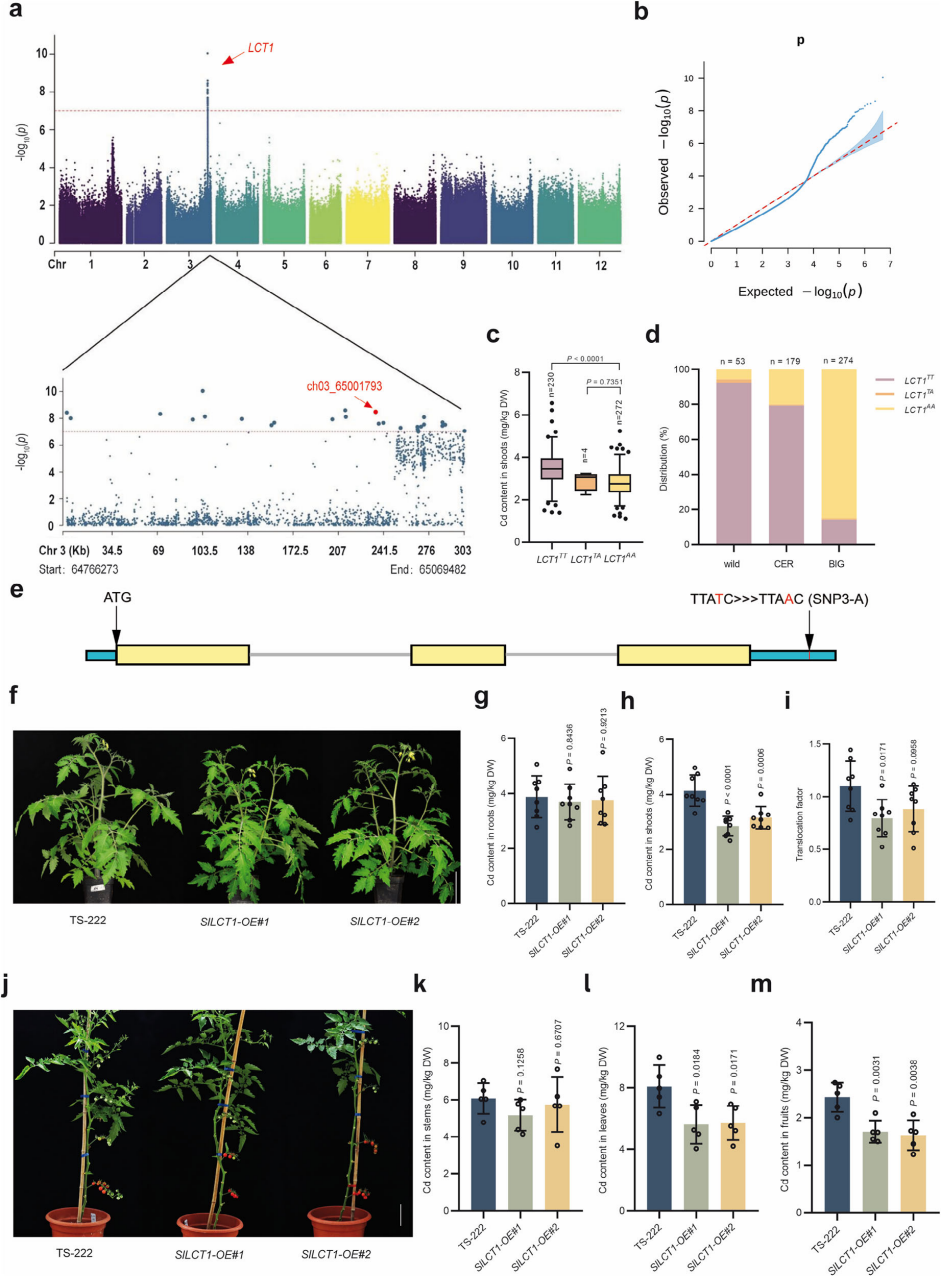
GWAS and functional analysis of *LCT1*. a) Manhattan plot of genome‐wide association study for Cd content in shoots. The lead SNP (ch03_65001793, *P* = 3.48 × 10^−9^) in the *LCT1* gene is indicated with a red arrow. b) Quantile–quantile plot for Cd content in shoots. c) Phenotypes of Cd content in shoots corresponding to three genotypes of *LCT1*. *P‐*values were determined by Student's *t*‐test, with *p* < 0.05 indicating a significant difference. d) Distribution of three *LCT1* genotypes across three tomato subgroups. e) Structure of the *LCT1* gene. The red font indicates the significant SNP associated with shoot Cd content, SNP3‐A. f) Growth of *LCT1‐OE* lines and the wild‐type TS‐222. Scale bar, 8 cm. g–i) Root Cd content (g), shoot Cd content (h), and Cd translocation factor (i) of wild type TS‐222 and *LCT1‐OE* lines after 30 d of 1.0 mg kg^−1^ CdCl_2_ treatment. j) Growth of wild‐type TS‐222 (Same as Figure S3l,j, Supporting Information), *LCT1‐OE#1*, and *LCT1‐OE#2* lines under 1.0 mg kg^−1^ CdCl_2_ treatment. Scale bar, 12 cm. k–m) Stem Cd content k), leaf Cd content l), and fruit Cd content m) in wild‐type TS‐222, *LCT1‐OE#1*, and *LCT1‐OE#2* lines (The data for TS‐222 are the same as those shown in Figure S3l–o, Supporting Information). *P‐*values were determined by Student's *t*‐test, with *p* < 0.05 indicating a significant difference.

The “Cd accumulation” trait has received relatively little attention during the process of tomato domestication and breeding. To understand why this trait underwent obvious selection, we investigated the genetic mechanisms behind low Cd accumulation in tomato. GWAS is an effective strategy for uncovering the genetic basis of ion accumulation in plants.^[^ ^30^, ^32^, ^33^, ^34^ ^]^ To identify the genetic alleles contributing to Cd accumulation, we performed a GWAS using 5 096 140 common SNPs with a minor allele frequency (MAF) > 0.05 and a missing ratio < 10%. A significance threshold of *p*‐values ≤ 1.0 × 10^−7^ was established following Bonferroni adjustment (**Figure** **2**a,b; Figure S2d–g, Supporting Information). A strong association signal, named *LCT1* (*Low Cadmium in Tomato 1*), was observed on chromosome 3 in the GWAS for Cd content in shoots, comprising 27 significant SNPs. We then analyzed the physical positions, genomic elements, and functional annotations surrounding the significant SNPs to select candidate genes underlying Cd content in shoots. The results showed that the majority of SNPs had no discernible effect (Table S3, Supporting Information). Notably, however, three significant SNPs were located within the promoter region (SNP15‐T), exon 2 (SNP16‐T), and 3’UTR (SNP3‐A) of the gene *Solyc03g115220* (Figure 2e; Table S3, Supporting Information). *Solyc03g115220* encodes a flavonoid 3’‐hydroxylase, belonging to the CYP450 family. The SNP15‐T, located within the promoter region, is predicted not to reside within any known cis‐regulatory elements, and it does not affect gene expression (Figure S2h–j, Supporting Information). SNP16‐T is a synonymous mutation. SNP3‐A is predicted to reside within the binding site of *miR8762*, which may disrupt its binding (Figure S2k,l; Table S3, Supporting Information).

Genotype analysis reveals that accessions with the 3’UTR^AA^ genotype (*LCT1^AA^*) and the heterozygous 3’UTR^TA^ genotype (*LCT1^TA^*) have lower Cd content compared to those with the 3’UTR^TT^ genotype (*LCT1^TT^*) (Figure 2c). This finding indicates a significant correlation between variation in the 3’UTR and Cd accumulation, with *LCT1^T^* classified as the high‐Cd allele and *LCT1^A^* as the low‐Cd allele. Additionally, we examined the distribution of genotypes across various tomato subspecies. We found that the wild and CER groups predominantly carried the *LCT1^TT^* genotype, accounting for 92.45% and 79.33% respectively, while the BIG group primarily exhibited the *LCT1^AA^* genotype at a frequency of 85.04% (Figure 2d). Hence, we focused on *LCT1* to understand its potential genetic mechanism underlying low Cd accumulation in tomato.

We hope to utilize *LCT1* to transform “high Cd” tomato into “low Cd”. We generated two tomato *LCT1* overexpression (*LCT1*‐OE) lines in the background of “high Cd” accession TS‐222 and confirmed their elevated *LCT1* RNA levels (Figure S3a, Supporting Information). We measured the Cd accumulation abilities of *LCT1*‐OE*#1* and *#2* lines together with TS‐222 and found that overexpressing *LCT1* in tomato reduced the Cd content in the shoots and fruits (Figure 2f–m). Additionally, a functional experiment was performed by using CRISPR/Cas9 to knock out *LCT1* in TS‐222 (Figure S3b, Supporting Information). Interestingly, the successful knockout of *LCT1* also reduced the Cd content in the shoots and fruits (Figure S3c–o, Supporting Information).

Through RNA‐Seq and metabolite analysis, we concluded that the similar phenotypes were possibly not due to cosuppression in the overexpression lines or compensatory effects from other genes in *lct1* mutants (Figures S4 and S5, Supporting Information). RNA‐seq revealed that genes involved in glutathione metabolism were significantly up‐regulated in the *LCT1‐OE* lines, whereas auxin‐signalling genes were markedly induced in the *lct1* mutants (Figure S4g–m, Supporting Information). Metabolite profiling showed that total flavonoid levels were elevated in both *LCT1‐OE* lines and *lct1* mutants (Figure S5a, Supporting Information). Specifically, quercetin accumulation was significantly higher in *LCT1‐OE* plants, while kaempferol content was markedly increased in *lct1* mutants (Figure S5b–f, Supporting Information). Therefore, we speculate the *LCT1‐OE* lines may primarily function through the chelation of Cd by quercetin and glutathione, whereas the *lct1* mutant may act through the chelation of Cd by kaempferol and the auxin signaling pathway. Thus, these findings collectively highlight the complex and multifaceted role of *LCT1* in regulating Cd accumulation in tomato, which has important implications for breeding low‐Cd tomato varieties.

### 2.3 *LCT1* Expression Variance: A Key to Low Cd in Cultivated Tomato

**Figure 3:**
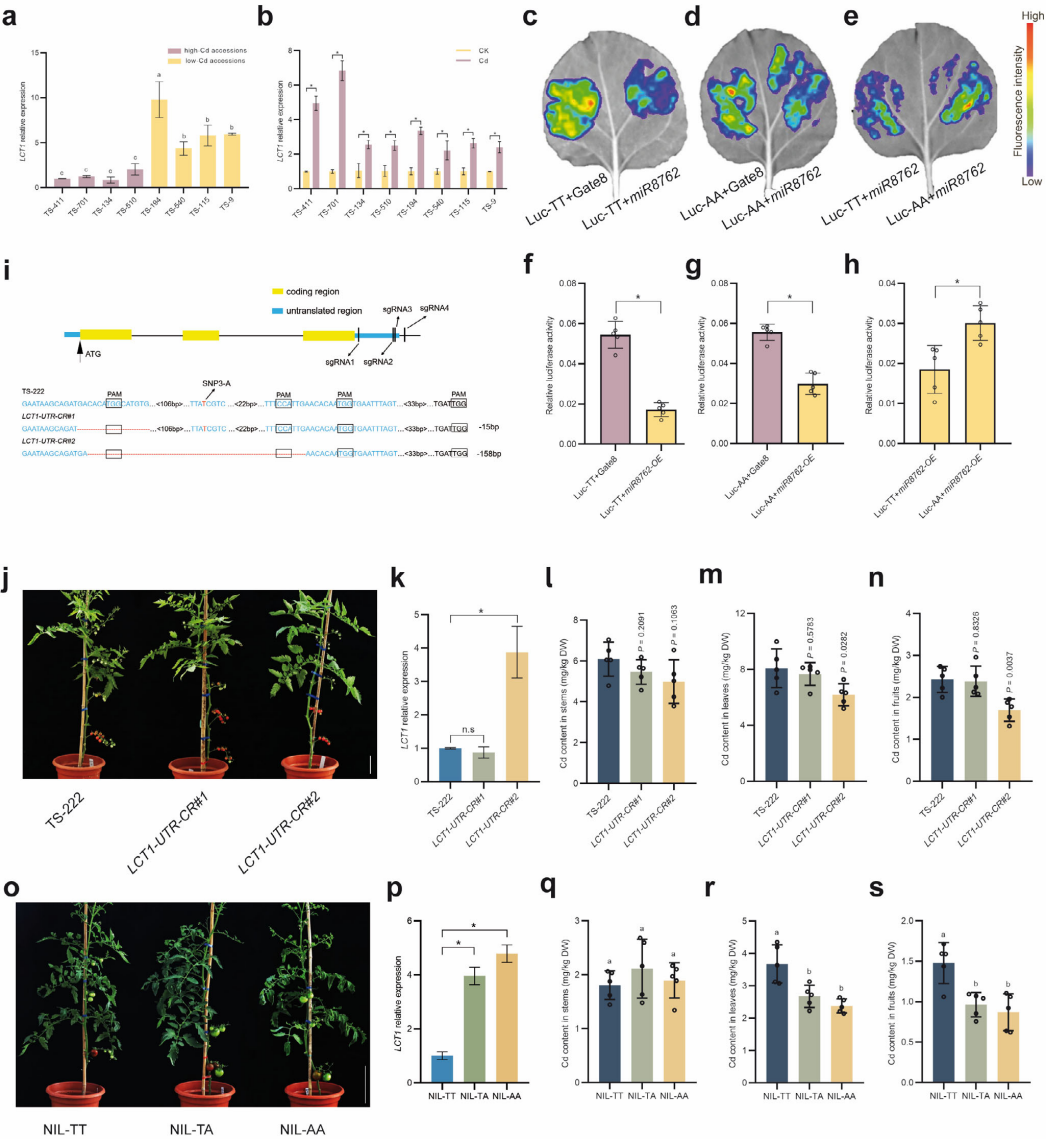
Regulation of *LCT1* expression and Cd accumulation in tomato by SNP3‐A. a) *LCT1* gene expression levels in high and low Cd‐accumulating tomato accessions. Different lowercase letters indicate significant differences (*p* < 0.05). b) Changes in *LCT1* gene expression levels in high and low Cd‐accumulating tomato accessions after 30 d of treatment with 0 mg kg^−1^ (CK) or 1.0 mg kg^−1^ CdCl_2_ (Cd). An asterisk (*) denotes significant difference (*p* < 0.05). c,f) The effect of *miR8762* on the fluorescence intensity (c) and enzymatic activity (f) of Luc‐TT. The left side shows co‐expression of Luc‐TT and the Gate8 control vector in tobacco leaves, while the right side shows co‐expression of Luc‐TT and *miR8762* in tobacco leaves. d,g) The effect of *miR8762* on the fluorescence intensity (d) and enzymatic activity (g) of Luc‐AA. The left side shows co‐expression of Luc‐AA and the Gate8 control vector in tobacco leaves, while the right side shows co‐expression of Luc‐AA and *miR8762* in tobacco leaves. e,h) Comparison of fluorescence intensity (e) and enzymatic activity (h) of Luc‐TT and Luc‐AA when co‐expressed with *miR876*2. i) The sequence of the wild‐type TS‐222 and the sequences of two mutants, *LCT1‐UTR‐CR#1* and *LCT1‐UTR‐CR#2*. The sgRNAs were designed using an online tool (http://skl.scau.edu.cn/) targeting four specific sites with the PAM motif of 5’‐NGG‐3’. j) Growth of wild‐type TS‐222 (Same as Figure 2j; Figure S3l, Supporting Information), *LCT1‐UTR‐CR#1*, and *LCT1‐UTR‐CR#2* under 1.0 mg kg^−1^ CdCl_2_ treatment. Scale bar, 12 cm. k) Relative expression levels of *LCT1* in *LCT1‐UTR‐CR* plants. l–n), Stems (l), leaves (m), and fruits (n) Cd content in wild‐type TS‐222, *LCT1‐UTR‐CR#1*, and *LCT1‐UTR‐CR#2* (The data for TS‐222 are the same as those shown in Figure 2k–m and Figure S3m–o, Supporting Information). *P‐*values were determined by Student's *t*‐test, with *p* < 0.05 indicating a significant difference. o Growth of NIL plants. Scale bar, 20 cm. p) Relative expression levels of *LCT1* in NILs plants. q–s) Stems (q), leaves (r), and fruits (s) Cd content in NILs after 1.0 mg kg^−1^ CdCl_2_ treatment. Different lowercase letters indicate significant differences (*p* < 0.05).

In our analysis of *LCT1* expression levels in tomato, we observed that “low Cd” accessions with *LCT1^AA^* genotype exhibited higher expression levels relative to “high Cd” accessions with *LCT1^TT^* genotype, and that Cd treatment induced the expression of *LCT1* (**Figure** **3**a,b). The 3’UTR region harboring the polymorphism was predicted to be a binding site for *miR8762* (Figure S2k–l, Supporting Information). Hence, employing the dual‐luciferase reporter assay system, we fused the two 3’UTR sequences (3’UTR^TT^ and 3’UTR^AA^) of *LCT1* with the luciferase gene sequence and co‐expressed them separately with *miR8762* in tobacco (*Nicotiana tabacum*) leaves (Figure S6, Supporting Information). The results indicated that *miR8762* exerted inhibitory effects on the luciferase fused with both types of 3’UTRs, but the luciferase construct fused with the 3’UTR^AA^ showed a weaker inhibition (Figure 3c–h). Therefore, the 3’UTR variation (SNP3‐A) of *LCT1* can alleviate the inhibitory effect of *miR8762* on *LCT1* expression.

To further confirm the impact of SNP3‐A on *LCT1* expression and Cd accumulation in tomato, we employed CRISPR/Cas9 to edit the 3’UTR of *LCT1*. We generated two lines, *LCT1‐UTR‐CR#1* and *LCT1‐UTR‐CR#2*, with deletions of 15 bp and 158 bp in the 3’UTR, respectively (Figure 3i). We found that, compared with TS‐222, the *LCT1‐UTR‐CR#2* line exhibited significantly higher *LCT1* expression levels and lower Cd accumulation in the shoots and fruits, while no significant changes were observed in the *LCT1‐UTR‐CR#1* line (Figure 3j–m). This discrepancy may be due to the editing site in *LCT1‐UTR‐CR#1* being too far from the binding site of *miR8762* (Figure 3i).

We next categorized the near‐isogenic lines (NILs) for *LCT1* into NIL‐TT, NIL‐TA, and NIL‐AA based on their genotypes (Figure S7, Supporting Information), and then compared their *LCT1* expression levels and Cd accumulation. Notably, both NIL‐TA and NIL‐AA exhibited higher *LCT1* expression levels than NIL‐TT (Figure 3p). Furthermore, compared to NIL‐TT, the leaf Cd content in NIL‐TA and NIL‐AA was reduced by 27.26% and 35.27%, respectively, while the Cd content in the fruits decreased by 34.82% and 41.16% (Figure 3r–s). Thus, the variation in the 3’UTR led to *LCT1* expression variance, thereby resulting in low Cd accumulation in cultivated tomato.

### 2.4 Co‐Selection of *LCT1* and Fruit Weight Locus *fw3.2*

The findings above have shown that the 3’UTR variation of *LCT1* is the genetic basis for Cd accumulation differences in tomato populations. Therefore, we used this variation as a clue to explore why the trait of “Cd accumulation” in tomato, which has received little attention from breeders, has undergone obvious selection, leading to current the low‐Cd accumulation phenotype in most cultivated tomatoes.

**Figure 4:**
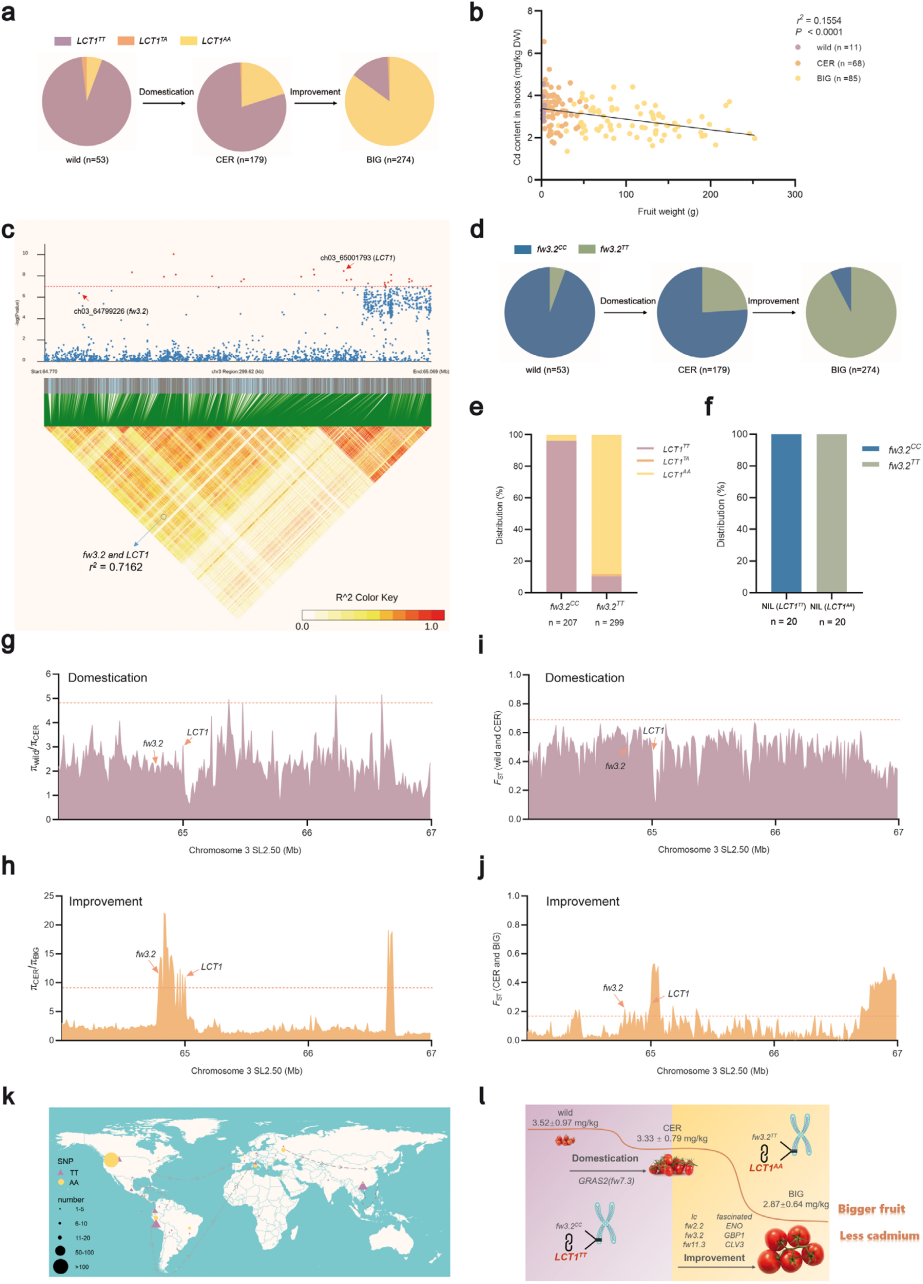
Co‐selection of *LCT1* and fruit weight locus *fw3.2*. a) Distribution of *LCT1* genotypes during the domestication and improvement of tomato. b) Linear regression between fruit weight and shoot Cd content in 164 tomato accessions. Data on fruit weight from 164 tomato accessions are sourced from a previous study.^[^ ^23^ ^]^ *r^2^*, coefficient of determination. N is the accession number of the three groups. c) Linkage disequilibrium analysis of the genomic segment from 64.77 to 65.07 Mb on chromosome 3. The red arrow on the left indicates the key SNP ch03_64799226 in the promoter of the tomato fruit weight gene *fw3.2*. The red arrow on the right indicates the key SNP ch03_65001793 in the 3’UTR of the tomato Cd accumulation gene *LCT1*. The blue arrow represents the correlation coefficient *r^2^* between ch03_64799226 and ch03_65001793; a value closer to 1 indicates a tighter linkage between the two loci. d) Distribution of the two *fw3.2* genotypes during the domestication and improvement of tomato. *fw3.2^CC^* represents the small‐fruit genotype, while *fw3.2^TT^* indicates the large‐fruit genotype. e) Combined analysis of *fw3.2* and *LCT1* genotypes in 506 tomato accessions. *LCT1^TT^* is the high‐Cd genotype, and *LCT1^AA^* is the low‐Cd genotype. f) Distribution of the two *fw3.2* genotypes in *LCT1* NILs. g) Ratio of nucleotide diversity (π) between the wild and CER subgroups (ch03_64.00–67.00 Mb). The red dashed line represents the top 5% threshold of the π ratio across the entire chromosome 3 between the wild and CER subgroups (π_wild_/π_CER_ ≥ 4.8053). h) Ratio of π between the CER and BIG subgroups (ch03_64.00–67.00 Mb). The red dashed line represents the top 5% threshold of the π ratio across the entire chromosome 3 between the CER and BIG subgroups (π_CER_/π_BIG_ ≥ 9.0313). The red arrow indicates the genomic locations of *fw3.2* and *LCT1*. i) Population differentiation statistic (*F*_ST_) between the wild and CER subgroups (ch03_64.00–67.00 Mb). The red dashed line represents the top 5% threshold of the *F*_ST_ across the entire chromosome 3 between the wild and CER subgroups (*F*_ST_ ≥ 0.6830). j) *F*_ST_ between the CER and BIG subgroups (ch03_64.00–67.00 Mb). The red dashed line represents the top 5% threshold of the *F*_ST_ across the entire chromosome 3 between the CER and BIG subgroups (*F*_ST_ ≥ 0.1634). k) Geographic distribution of tomato accessions with the two *LCT1* genotypes. The dashed arrow represents the spread path of the tomato. l) Model of the co‐selection of fruit weight and low Cd trait during the domestication and improvement of tomato.

We found that the majority of accessions in the wild group belong to the *LCT1^TT^* genotype, while most of the accessions in the BIG group had the *LCT1^AA^* genotype (**Figure** **4**a). Throughout the domestication and improvement of tomato, there has been a gradual increase in the prevalence of the *LCT1^AA^* genotype, particularly during the transition from CER to BIG, where it rose from 20.67% to 85.77% (Figure 4a). This is consistent with the phenotypic trends observed in the three groups (Figure 1e). We hypothesized that this phenomenon may be attributed to genetic hitchhiking, since there is no record of targeted selection for Cd accumulation traits during the tomato domestication and improvement process. Thus, our focus turned to fruit weight, a trait closely linked to yield, which is a major target for breeders. A significant negative correlation between fruit weight and Cd content in shoots was found among the 164 tomato accessions (Figure 4b), providing preliminary validation for our hypothesis.

For genetic hitchhiking to occur, it is essential that two loci are located on the same chromosome and separated by a relatively short genetic distance.^[^ ^35^, ^36^ ^]^ In our study, we investigated QTLs associated with yield and identified a QTL, *fw3.2*, on chromosome 3 that controls fruit size, with a highly correlated SNP (ch03_64799226) in its promoter region^[^ ^37^, ^38^ ^]^ (Table S4, Supporting Information). The linkage disequilibrium (LD) analysis revealed a tight linkage between the SNP (ch03_64799226) in the promoter of *fw3.2* and the SNP (ch03_65001793) in the 3’UTR of *LCT1* (Figure 4c). We examined the distribution of *fw3.2^CC^* and *fw3.2^TT^* alleles in the wild, CER, and BIG accessions. The prevalence of the *fw3.2^TT^* allele, representing large fruit, gradually increased within the population, notably rising from 24.02% to 92.34% during the transition from CER to BIG (Figure 4d). This trend closely paralleled changes in the prevalence of the *LCT1* within the population (Figure 4a). Accessions carrying the *fw3.2^CC^* allele mostly co‐carried the *LCT1^TT^* allele, associated with “high Cd”, whereas accessions carrying the *fw3.2^TT^* allele predominantly co‐carried the *LCT1^AA^* allele, linked to “low Cd” (Figure 4e; Figure S8a, Supporting Information). Additionally, the two genotypes of *fw3.2* neither affect Cd accumulation in tomato nor are induced by Cd exposure (Figure S8, Supporting Information). Furthermore, analysis of near‐isogenic lines (NILs) of *LCT1* indicated that all accessions of the NIL (TT) genotype contain the *fw3.2^CC^* allele, while all accessions of the NIL (AA) genotype contain the *fw3.2^TT^* allele (Figure 4f).

We then assessed the nucleotide diversity (π) and the population differentiation statistic (*F*_ST_) for the region surrounding these two genes. Comparing the π and *F*_ST_ between CER and BIG accessions, we found that the π_CER_/π_BIG_ and *F*_ST_ for *fw3.2* and *LCT1* exceeded the top 5% threshold for chromosome 3 (Figure 4g–j). This suggests that during the improvement process from CER to BIG, *fw3.2* and *LCT1* underwent significant selection pressure. Additionally, *fw3.2* and *LCT1* appear to reside within a large genomic cluster, further supporting the notion that they have undergone similar selective pressures during the improvement process from CER to BIG in tomato (Figure 4h,j). Furthermore, the geographical distribution indicates that *LCT1^TT^* is mainly distributed in regions where tomato originate, such as Peru and Ecuador, whereas *LCT1^AA^* is mainly found in the United States, Russia, and along the Mediterranean coast (e.g., in Italy) (Figure 4k; Table S5, Supporting Information). Thus, we proposed that the “low Cd” genotype *LCT1^AA^* has been retained in tomato during long‐term domestication, and underwent strong selective pressure by genetic hitchhiking with *fw3.2^TT^* during the breeding process from CER to BIG. These events have led to the dominance of the “low Cd” genotype *LCT1^AA^* in cultivated tomato today (Figure 4k,l).

### 2.5 Mechanistic Insights into *LCT1*‐Mediated Cd Reduction in Tomato

**Figure 5:**
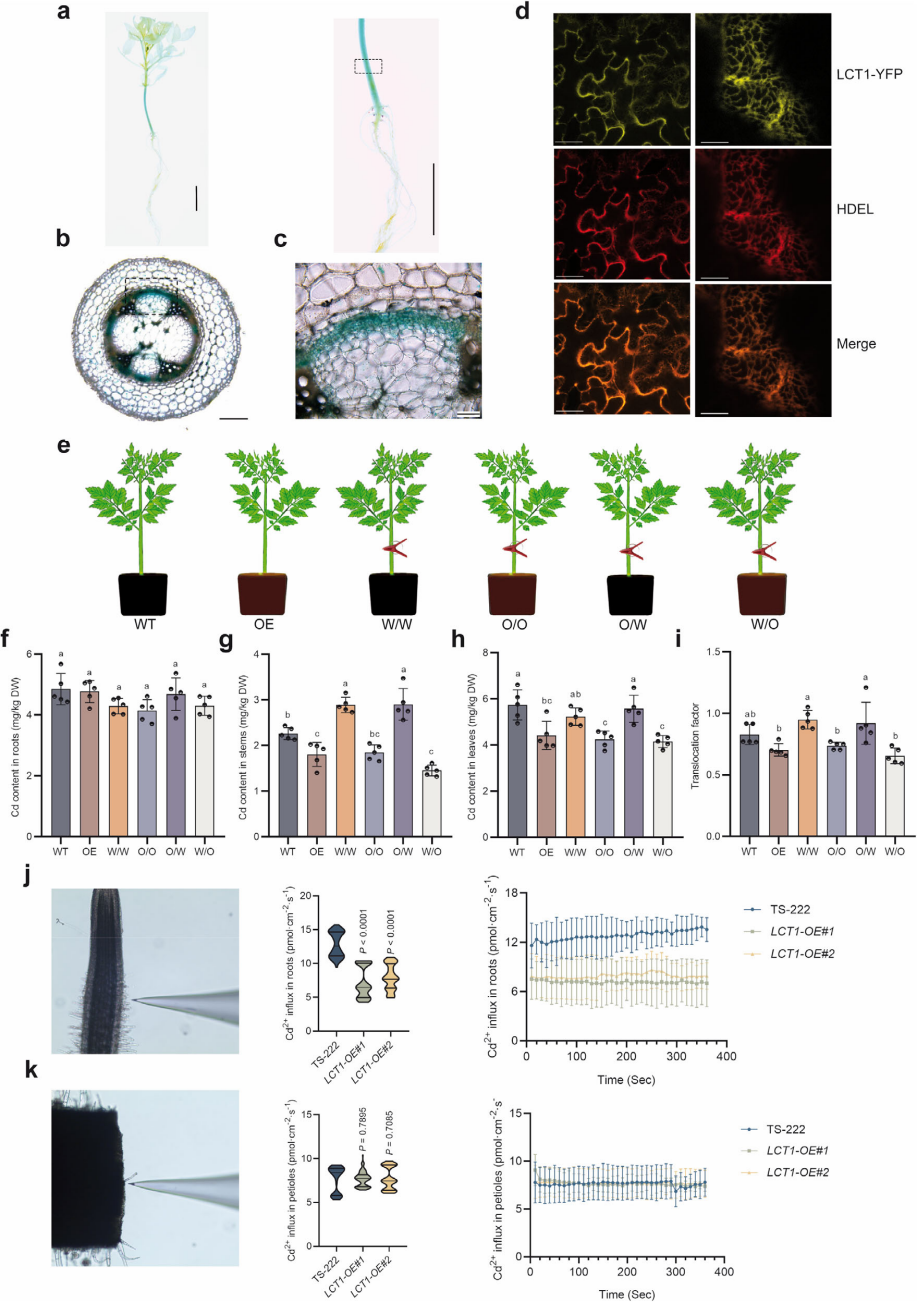
Mechanistic insights into *LCT1*‐mediated Cd reduction in tomato. a) The GUS activity was examined in seedlings of 30‐day‐old *LCT1pro*: *GUS* transgenic tomato (left), and the amplified image of the hypocotyl (right). Scale bars, 2 cm. The dashed box indicates the part to be used for slicing (b). Scale bars, 2.5 cm. b) GUS activity examined in the cross‐sections of the hypocotyl. Scale bars, 400 µm. c) The amplified image of cross‐sections from (b) (indicated by dashed box). Scale bars, 100 µm. d) Subcellular localization of *LCT1*‐YFP in tomato. YFP, yellow fluorescence protein. HDEL, a red fluorescent marker of the endoplasmic reticulum. Scale bars, 30 µm (left) and 8µm (right). e) Schematic diagram of grafting. WT denotes wild‐type TS‐222. OE signifies *LCT1‐OE* lines. W/W represents self‐graft of wild‐type TS‐222. O/O indicates self‐graft of *LCT1‐OE* lines. O/W signifies grafting with TS‐222 as the rootstock and *LCT1‐OE* lines as the scion. W/O denotes grafting with *LCT1‐OE* lines as the rootstock and wild‐type TS‐222 as the scion. f–i) Cd content in roots (f), stems (g), and leaves (h), and Cd translocation factor (i) for the six combinations. Different lowercase letters indicate significant differences (*p* < 0.05). j) Cd^2+^ influx rate in the root of wild‐type TS‐222 and *LCT1‐OE* lines. k) Cd^2+^ influx rate in the petiole of wild‐type TS‐222 and *LCT1‐OE* lines. *P‐*values were determined by Student's *t*‐test, with *p* < 0.05 indicating a significant difference.

These findings above explain why large‐fruited tomatoes predominantly exhibit low‐Cd accumulation. However, it remains crucial to understand how F3’H, encoded by *LCT1*, reduces Cd accumulation. Therefore, we further explored the mechanisms by which *LCT1* reduces Cd accumulation. Promoter‐GUS analysis revealed that *LCT1* is primarily expressed in the tomato hypocotyl (**Figure** **5**a), and subcellular localization indicated that F3’H is mainly located on the endoplasmic reticulum membrane (Figure 5d). In hypocotyl cross‐sections, GUS activity was primarily detected in the vascular bundle sheath (Figure 5b,c).

We conducted grafting experiments using the hypocotyl as the junction point. The results showed that *LCT1‐OE* effectively reduces Cd accumulation in tomato when serving as the rootstock (Figure 5g–i). Conversely, when employed as the scion, *LCT1‐OE* does not reduce Cd accumulation in tomato (Figure 5g–i). This implies that *LCT1* reduces Cd accumulation in tomato primarily through regulation in the parts below the hypocotyl, rather than through regulation in the aerial parts.

We then employed the Noninvasive Micro‐test Technique (NMT) to measure Cd^2+^ influx in the roots and petioles of wild‐type TS‐222 and *LCT1‐OE* plants (Figure 5j,k). The results indicated that Cd^2+^ uptake in the roots of *LCT1‐OE* plants was significantly lower than that of WT, with reductions of 44.22% and 38.16%, respectively (Figure 5j). Conversely, no significant difference in Cd^2+^ influx was observed in the petioles between *LCT1‐OE* plants and WT (Figure 5k). Collectively, these findings suggest that *LCT1* primarily reduces Cd accumulation in the shoots and fruits by regulating Cd uptake in the roots, rather than by directly regulating Cd distribution in the shoots.

### 2.6 F3’H: A Universal Strategy for Cd Accumulation Reduction in Plants

F3’H is widely present in most plants and exhibits high similarity in the regulation of flavonoid synthesis pathways. However, whether its function in Cd accumulation regulation varies across different plants remains unclear. Therefore, we further validated its effect on reducing Cd accumulation in *Arabidopsis* and rice. We examined the genes encoding F3’H in *Arabidopsis* and *japonica* rice, namely *AtCYP75B1* (*AT5G07990*) in *Arabidopsis* and *OsCYP75B2* (*Os10g0320100*) and *OsCYP75B4* (*Os10g0317900*) in rice, which are orthologs of *LCT1*.

**Figure 6:**
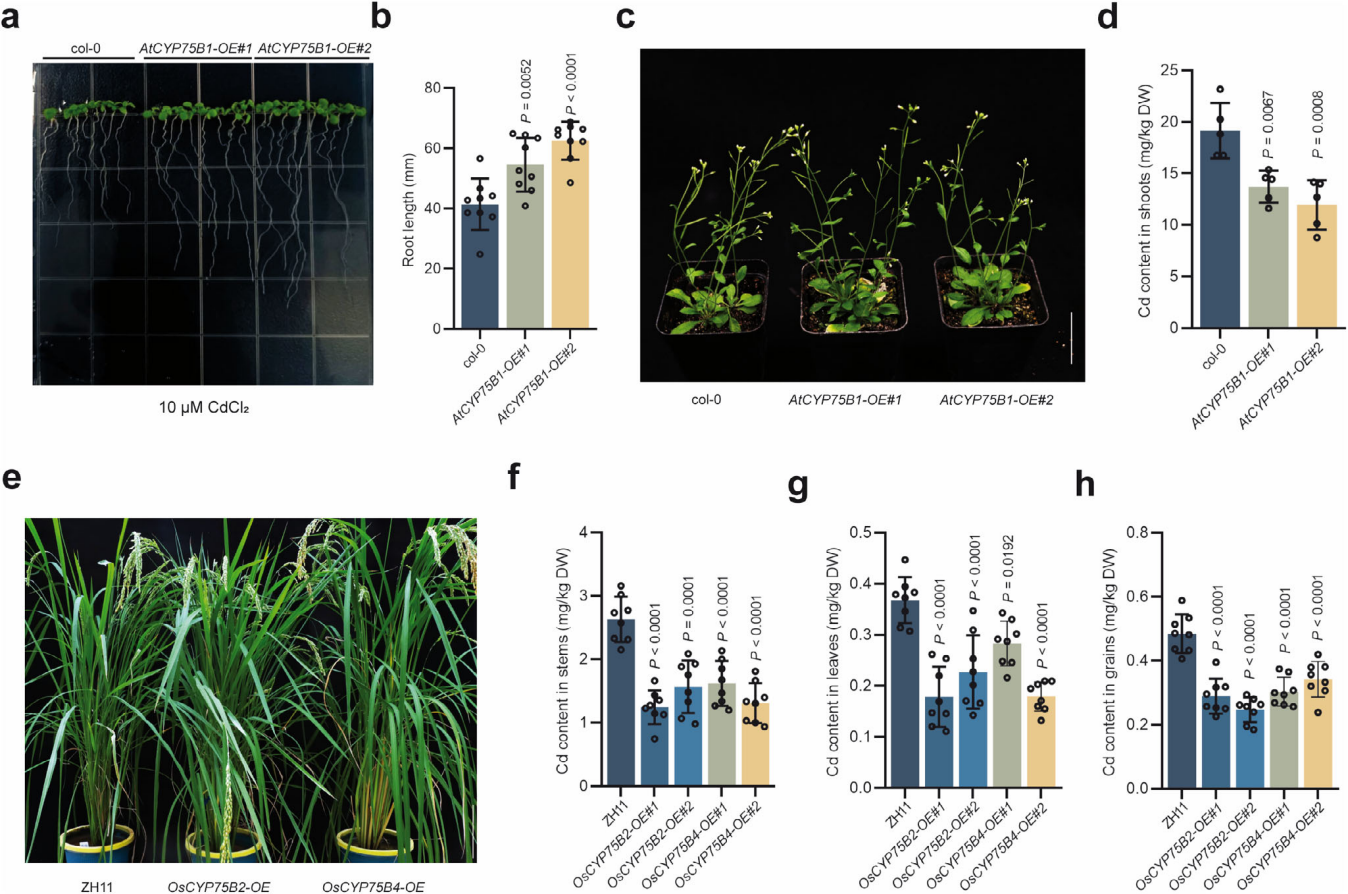
Role of *LCT1*’s orthologs *AtCYP75B1*, *OsCYP75B2*, and *OsCYP75B4* in Cd accumulation in *Arabidopsis* and rice. a,b) Root length of wild‐type Col‐0, and *AtCYP75B1‐OE* lines after 14 d of growth on 1/2 MS medium supplemented with 10 µm CdCl_2_. c,d) Growth and shoot Cd content of potted wild‐type Col‐0 and *AtCYP75B1‐OE* lines after 30 d of 1.5 mg kg^−1^ CdCl_2_ treatment. e) Growth of potted rice wild‐type ‘ZH11’, *OsCYP75B2‐OE* line, and *OsCYP75B4‐OE* line in soil with a Cd concentration of 0.8 mg kg^−1^. Scale bar, 20 cm. f–h) Cd content in leaves (f), stems (g), and grains (h) of potted rice wild‐type ‘ZH11’, *OsCYP75B2‐OE*, and *OsCYP75B4‐OE* lines. *P‐*values were determined by Student's *t*‐test, with *p* < 0.05 indicating a significant difference.

Under 10 µm Cd treatment in 1/2 MS medium, the root lengths of the *Arabidopsis* mutant *cyp75b1* and the *AtCYP75B1‐OE* plants were significantly greater than those of the wild‐type Col‐0 (**Figure** **6**a,b; Figure S9c,d, Supporting Information). After 30 d in a substrate containing 1.5 mg kg^−1^ Cd, the *AtCYP75B1‐OE* plants exhibited reductions in shoot Cd accumulation of 28.32% and 37.57%, while the *cyp75b1* mutant showed a 29.68% reduction compared to the WT (Figure 6c,d; Figure S9e,f, Supporting Information). These findings are consistent with observations in tomato, again indicating a complex role for F3’H in regulating Cd accumulation in plants. In rice, under pot conditions with a Cd concentration of 0.8 mg kg^−1^, the *OsCYP75B2‐OE* and *OsCYP75B4‐OE* plants exhibited reductions in Cd content of 23% to 51% in leaves, 38% to 50% in stems, and 29% to 49% in grains compared to the wild‐type ‘ZH11’ (Figure 6e–h). Thus, these results suggest that the function of F3’H in reducing plant Cd accumulation is relatively conserved across different species. This could potentially provide a solution to the current issue of excessive Cd in rice production.

## 3 Discussion

In the process of targeted selection for crop yield, it is often regrettable that numerous genes are lost, leading to a series of threats faced by global food production, such as salt, cold, and drought stress.^[^ ^30^, ^39^, ^40^ ^]^ Researchers had proposed strategies such as distant hybridization, induced mutation, and *de novo* domestication to address these challenges.^[^ ^41^, ^42^, ^43^ ^]^ It is noteworthy that retention of “invisible traits” during plant domestication is not always an unfortunate outcome; the “low Cd” trait highlighted in this study serves as a fortunate example. Another, from previous research, is the significant increases in nicotinic acid and succinyladenosine during tomato domestication and improvement processes, metabolites with benefits to both human health and plant growth^[^ ^27^ ^]^ (Figure S10a,b, Supporting Information). Evolutionary analysis indicated that the leading SNPs regulating these metabolites were found to be in the region associated containing the fruit weight gene *fw11.3* and associated with an improvement sweep^[^ ^27^ ^]^ (Figure S10c,d, Supporting Information). These findings suggest that there are still many fortunate “invisible traits” and identification of their major QTLs awaits further exploration.

**Figure 7:**
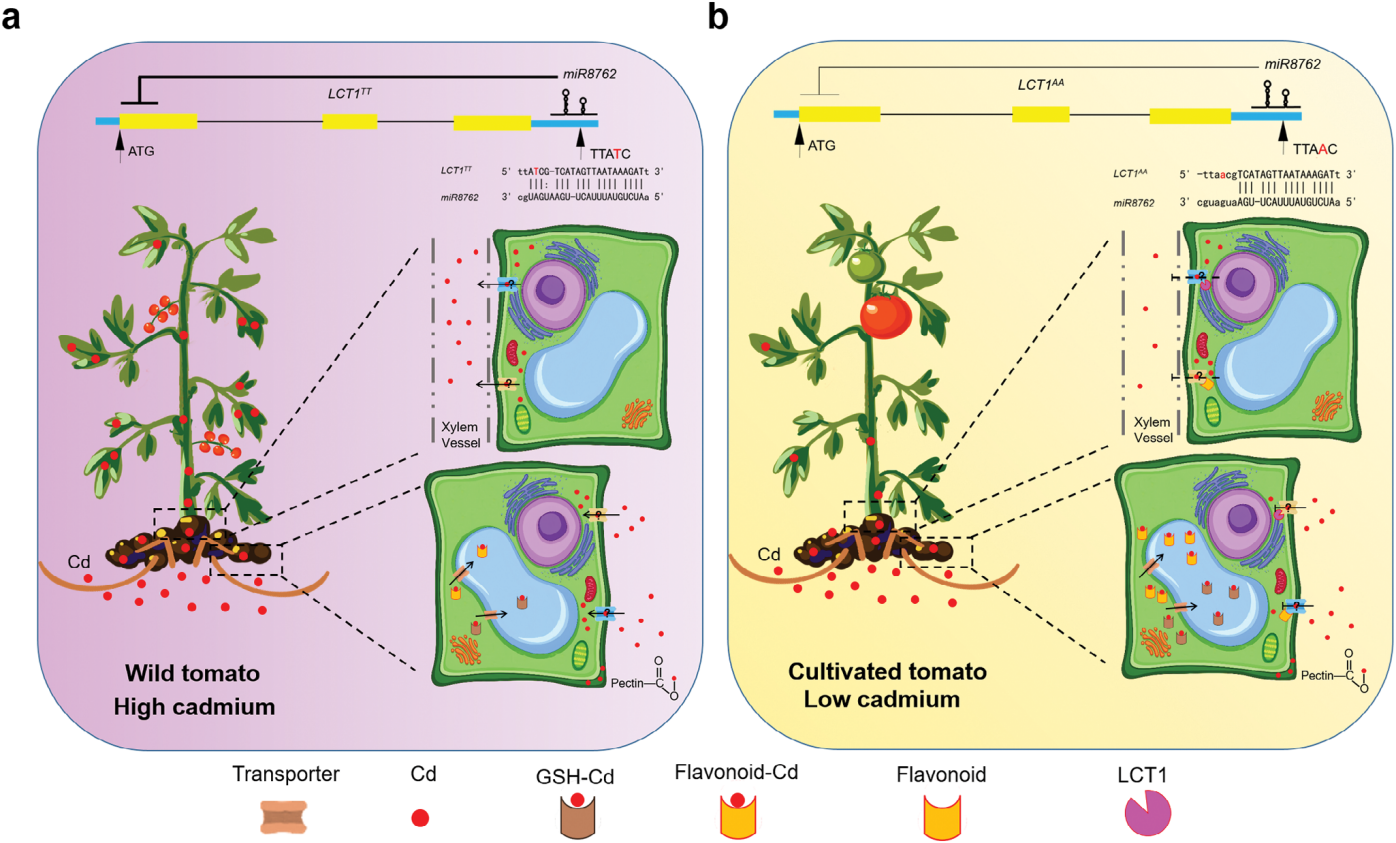
A model illustrating how the *LCT1* reduced Cd accumulation in cultivated tomato. a) In wild tomato, *LCT1* is strongly inhibited by *miR8762*, resulting in low expression levels. Consequently, the synthesis of metabolites such as flavonoids and glutathione is limited, leading to weaker chelation with Cd. Additionally, the roots of wild tomato exhibit a stronger capacity to absorb Cd, resulting in higher Cd accumulation in the aerial parts of the plant. b) In cultivated tomato, *LCT1* is less inhibited by *miR8762* and exhibits higher expression levels. This promotes the synthesis of flavonoids, glutathione, and other metabolites, which enhances Cd chelation. Meanwhile, this process reduces Cd uptake by the roots, leading to lower Cd accumulation in the aerial parts.

It is noteworthy that overexpressing *LCT1* also reduced Cd absorption by the roots (Figure 5j). This suggests that flavonoid 3’‐hydroxylase not only regulates Cd accumulation through chelation pathways but may also contribute to regulating Cd accumulation by inhibiting its uptake and translocation. One possibility is that F3’H interacts with specific Cd transporters to modulate Cd uptake, as suggested by a previous study^[^ ^44^ ^]^ (**Figure** **7**). Alternatively, akin to how small‐molecule drugs target ion channels in medicine,^[^ ^45^, ^46^ ^]^ flavonoids regulated by *LCT1* may interact with specific transporters to regulate Cd uptake (Figure 7). Our result suggests that F3’H may interact with ABC family transporters (Figure S11 and Table S6, Supporting Information), consistent with previous findings that cytochrome oxidase interacts with transporters to regulate ion transport.^[^ ^44^ ^]^ However, the precise mechanism by which F3’H interacts with ABC transporters to regulate Cd transport remains to be elucidated. Furthermore, it is worth exploring whether flavonoids target transporters to influence Cd uptake.

In summary, we have identified a quantitative trait locus for “low Cd” in tomato, named *LCT1*. Furthermore, we provided an explanation for why cultivated tomato today mainly exhibit “low Cd” traits, which is attributed to the genetic hitchhiking and co‐selection between the low‐Cd allele *LCT1^A^* and the large‐fruit allele *fw3.2^T^*. Currently, we can utilize existing varieties with “low Cd” genotypes (*LCT1^AA^)* to improve some major cultivars with “high Cd” genotypes (*LCT1^TT^*) to produce healthier and safer tomatoes. The conserved function of *LCT1* and its orthologues in *Arabidopsis* and rice makes a brighter prospect in crop improvement.

## 4 Experimental Section

### Plant Material and Growth Conditions

The 506 tomato accessions used for the GWAS were sown and harvested between October and November 2020 in a plastic greenhouse at Huazhong Agricultural University (30°27’N, 114°20’E). Following the Chinese agricultural land soil pollution risk control standard (GB 15618‐2018) and Chinese soil pollution standards, a substrate composed of peat, perlite, and vermiculite was treated with CdCl_2_·2.5H_2_O to achieve a mild Cd pollution level of 1.0 mg kg^−1^. After thorough mixing, the substrate was transferred to planting troughs. Post‐germination, the 506 tomato accessions were directly sown into the Cd‐treated substrate. After emergence, at least five uniform seedlings per accession were retained and appropriately watered. After 30 d of growth, root and shoot samples were collected to measure root length, plant height, fresh weight, dry weight, and Cd content in both roots and shoots.

For the functional characterization of *LCT1* during the seedling stage, wild‐type TS‐222, *LCT1‐OE* lines, and *lct1* mutants were grown in a growth chamber at Huazhong Agricultural University. *LCT1‐OE* positive seedlings were screened by spraying kanamycin, PCR detection with the vector‐specific primers 35S and gate8‐Rv (Table S7, Supporting Information), and qRT‐PCR. The *LCT1* homozygous mutants were identified through vector detection with the specific primers pTX‐Fw and pTX‐Rv, PCR amplification with the specific primers LCT1‐JC‐Fw and LCT1‐JC‐Rv (Table S7, Supporting Information), and subsequent sequencing. At 14 d’ post‐germination, *LCT1‐OE* lines and *lct1* mutants, along with wild‐type TS‐222, were transplanted into a substrate composed of peat, perlite, and vermiculite with a Cd concentration of 1.0 mg kg^−1^. Eight biological replicates were set up and maintained appropriate water and nutrient management. The growth conditions were set at a 12 h light/12 h dark cycle with a temperature of 25 °C. After 30 d of growth, root and shoot samples were collected. The roots were washed sequentially with municipal water and ddH_2_O until the substrate was completely removed. Both roots and shoots were dried at 75 °C to a constant weight, and the Cd content in the roots and shoots was measured.

For the functional characterization of *LCT1* during the mature plant stage, the following materials were grown in a plastic greenhouse at Huazhong Agricultural University: wild‐type TS‐222 (The Cd accumulation control for *LCT1‐OE* lines, *lct1* mutants, and *LCT1‐UTR‐CR* lines), selected positive *LCT1‐OE* lines, *lct1* mutants, *LCT1‐UTR‐CR* positive plants, and genotyped NIL plants. At 30 d post‐germination, seedlings were transplanted into a substrate composed of peat, perlite, and vermiculite, treated with 1.0 mg kg^−1^ CdCl_2_. Five biological replicates were set up for each material. Regular watering, fertilizer management, and pruning were carried out until fruit maturity. Samples were subsequently collected to measure fruit weight, Cd content, and other relevant parameters.

For *Arabidopsis thaliana*, wild‐type Col‐0, screened mutant *cyp75b1*, and *AtCYP75B1‐OE* lines were germinated, and ten uniform seedlings were transferred to 1/2 MS medium supplemented with 10 µm CdCl_2_. Growth conditions were set to a 12 h light/12 h dark cycle at 25 °C. After 14 d, root lengths were recorded. In pot experiments, five uniform positive seedlings were transplanted into a substrate composed of peat, perlite, and vermiculite, treated with 1.5 mg kg^−1^ CdCl_2_, and grown in a growth chamber at Huazhong Agricultural University, under controlled conditions (12 h light/12 h dark cycle, 25 °C). After 30 d, the shoot dry biomass and Cd content were measured.

For rice, wild‐type ‘ZH11’, *OsCYP75B2‐OE*, and *OsCYP75B4‐OE* lines were grown in pots within a greenhouse at the Huazhong Agricultural University. The growth conditions were maintained at a 10 h light/14 h dark cycle, with temperatures ranging from 28 to 34 °C. After three weeks of growth, wild‐type ‘ZH11’ and overexpression lines seedlings were transplanted to Cd‐contaminated paddy soil (0.8 mg kg^−1^) and cultivated in the greenhouse until grain maturity. Samples of stems, leaves, and grains were collected to determine Cd content.

### Measurement of Cd^2+^ Contents

After harvesting, the samples were initially dried at 110 °C for 15 min and then dried to a constant weight at 75 °C. The dried samples were ground and sieved through a 0.149 mm nylon mesh for chemical analysis. Samples of 0.2–0.4 g were digested with 10 mL of nitric acid using a MARS6 microwave at a temperature gradient of 120–180 °C for 1 h. Following digestion, the samples were further heated in a high‐temperature metal bath at 180 °C to evaporate excess HNO_3_ until the liquid volume in the digestion tube was reduced to 1–2 mL. After cooling to room temperature, the samples were made up to 25 mL with deionized water (ddH_2_O) to prepare the stock solution. Depending on the sample type, the solutions were diluted 5–10 fold and analyzed for Cd content using an Agilent graphite furnace (Agilent AA 240Z), with a detection limit of 0.019 µg L^−1^. The translocation factor (TF) was defined as the ratio of Cd content in shoots to that in roots.^[^ ^47^ ^]^

### Genome‐Wide Association Study

A linear mixed model (LMM) was employed within the TASSEL software suite, using 5,096,140 SNPs (MAFs > 0.05, missing ratio < 10%) to perform GWAS for Cd content and TF in 506 accessions. A significance threshold of *P*‐values ≤ 1.0 × 10^−7^ was established following Bonferroni adjustment. The physical positions of the SNPs were identified using the tomato genome sequence, version SL2.50 (http://solgenomics.net). The results were visualized using CMplot software.^[^ ^48^ ^]^

### Gene Expression Analysis

The primary leaves of tomato and rice plants, as well as the rosette leaves of *Arabidopsis*, were used for RNA extraction. Total RNA was extracted using TRIzol reagent (RN0102, Aidlab, China). First‐strand cDNA was synthesized using HiScript II 1st Strand cDNA Synthesis Kit (+gDNA wiper) (R212‐01, Vazyme, China), following the manufacturer's instructions. Gene expression levels were quantified using quantitative real‐time PCR (qRT‐PCR) conducted on a QuantStudioTM 6 Flex System (ABI, USA). The tomato actin gene (*Solyc11g005330*), the rice actin gene (*Os11g0163100*), and the *Arabidopsis* actin gene (*AT3G18780*) were used as internal controls. Primer sequences (designed with Primer Premier 5) are listed in Table S7 (Supporting Information).

### Generation of Transgenic Plants

The full‐length coding sequence and cDNA sequence (including the TT genotype 3’UTR) of *SlLCT1* were amplified from TS‐222 cDNA and then cloned into pHELLSGATE8 downstream of the CaMV35S promoter to generate the overexpression constructs *35Spro:CDS* (*SlLCT1‐OE#1*) and *35Spro:cDNA* (*SlLCT1‐OE#2*). sgRNAs were designed using the CRISPR‐GE website (http://skl.scau.edu.cn). For the *lct1* mutants, sgRNA1 and sgRNA2 targeted two sites within the second exon of *LCT1*. For the *LCT1‐UTR‐CR* lines, sgRNA1, sgRNA2, sgRNA3, and sgRNA4 targeted four sites near critical SNPs in the 3’UTR of *LCT1*. The CRISPR/Cas9 system with the binary pTX vector was employed to construct the gene editing vectors for *LCT1*. The plasmids were introduced into the wild‐type tomato with high‐Cd TS‐222 plants using *Agrobacterium*‐mediated transformation.^[^ ^49^ ^]^

Using the Ensembl Plants genome database (http://plants.ensembl.org/), BLAST searches were used to identify orthologs of *SlLCT1* in *Arabidopsis* and *rice*: *AtCYP75B1* (*AT5G07990*), *OsCYP75B2* (*Os10g0320100*), and *OsCYP75B4* (*Os10g0317900*). The corresponding gene sequences were then downloaded. The CDS of *AtCYP75B1* was amplified from Col‐0 cDNA and cloned into the pHELLSGATE8 vector to generate the overexpression construct *35S:AtCYP75B1*. The plasmid was introduced into the wild‐type *Arabidopsis* Col‐0 plants using *Agrobacterium*‐mediated transformation by the floral dip method.^[^ ^50^ ^]^ The CDS sequences of rice *OsCYP75B2* and *OsCYP75B4* were amplified from ‘ZH11’ cDNA and cloned into the PU1300 vector, which was driven by the CaMV35S promoter, to produce the overexpression constructs *35S:OsCYP75B2* and *35S:OsCYP75B4*. The plasmids were introduced into the wild‐type rice ‘ZH11’ plants using *Agrobacterium*‐mediated transformation.

### RNA Sequencing

Primary leaves from wild‐type tomato (TS‐222), *LCT1‐OE* lines, and *lct1* mutants were collected with three biological replicates each and immediately frozen in liquid nitrogen. Total plant RNA was extracted, and the quality of the RNA samples was assessed before sending them to Personalbio Corporation (Shanghai, China) for RNA‐seq analysis. High‐quality clean reads were mapped to the reference genome (version SL2.50). Differentially expressed genes (DEGs) were identified for those that had a more than twofold change in expression level with *a p* value of < 0.05.

### Determination of Total Flavonoids

Total flavonoids were quantified with a commercial Plant Flavonoid Assay Kit (microplate method; Shanghai Enzyme‐linked Biotechnology Co., Ltd., China, Cat. No. ml076373) according to the manufacturer's instructions. Briefly, 0.02 g of freeze‐dried powder was extracted in 2 mL 60% (v/v) ethanol for 3 h at 60 °C under agitation. The extract was centrifuged at 10 000 rpm for 10 min at 25 °C. After centrifugation, the supernatant (100 µL) was mixed with 10 µL reagent A at room temperature for 6 min. Then 10 µL of reagent B were added and mixed for another 6 min. Finally, 80 µL reagent C were added. After reaction for 15 min, the absorbance at 510 nm was measured using a Synergy™ H1 hybrid multimode microplate reader (BioTek, Winooski, VT, USA). Rutin was used as a reference. The calibration curve was calculated as y  =  2.4474x ‐ 0.0237 with *R^2^*  =  0.9998.

### Flavonoid Profiling

Mature leaves from wild‐type tomato (TS‐222), *LCT1‐OE* lines, and *lct1* mutants were collected with three biological replicates each and immediately frozen in liquid nitrogen. For extraction, 200 mg of ground leaf tissue was weighed into a 2 mL centrifuge tube and mixed with 1.2 mL of 70% ethanol/water (v/v). The mixture was vortexed for 30 s, sonicated for 30 min at 4 °C, vortexed again for 30 s, centrifuged (12 000 rpm, 10 min, 4 °C), and filtered through a 0.22‐µm nylon membrane before analysis with the UPLC Q‐Orbitrap system.

Liquid chromatography was performed using an Agilent InfinityLab Poroshell 120 EC‐C18 column (100 × 2.1 mm, 2.7 µm) at a flow rate of 0.3 mL min^−1^ and a temperature of 40 °C. The mobile phase comprised 0.1% formic acid in water (Solvent A) and 0.1% formic acid in acetonitrile (Solvent B). The linear gradient elution was programmed as follows: 0–1.0 min, 97% A; 1.0–18.0 min, 97% A to 10% A; 18.0‐20.0 min, 10% A; 20–20.1 min, 97% A; 20.1–23 min, 97% A. The injection volume was set at 5 µL. Sample detection commenced once instrument stability was achieved.

The Q‐Exactive mass spectrometer was configured with an ESI ion source, operating at 3500 V in both positive and negative modes. Data acquisition was conducted in centroid mode, featuring a full spectrum scan resolution of 70 000 and a scan range of 100–1000 *m z* ^−1^. Data Dependent Acquisition was employed for MS/MS metabolite information, with collision energies set at 15, 30, and 45 V. Chromatographic data were analyzed using Xcalibur 3.1 software.

Identification databases used included mzCloud and ChemSpider. Heatmaps were analyzed using TBtools‐IIv1.120.

### Western Blot

To confirm the knockout of *LCT1* in the *lct1* mutants, protein analysis was performed. The coding sequences of *lct1‐1* and *lct1‐2* mutants, as well as the wild‐type *LCT1* from TS‐222, were cloned into the pHELLSGATE8 vector containing a 6 × FLAG tag to generate fusion proteins. These constructs were transiently expressed in tobacco leaves. Western blot analysis was employed to detect *LCT1* protein levels before and after mutation, with Actin serving as the internal control.

### Development of Near‐Isogenic Lines for *SlLCT1*

Parental lines with extreme Cd accumulation phenotypes, TS‐540 (low Cd, genotype *LCT1^AA^*) and TS‐411 (high Cd, genotype *LCT1^TT^*), were crossed. TS‐540 served as the recurrent parent for consecutive backcrosses. During this process, individuals with the *LCT1^TA^* genotype were selected using the *LCT1*‐CAPS molecular marker, which was designed based on SNP3‐A in the 3’UTR of *LCT1*. After three backcrosses and one subsequent self‐cross, NILs for *LCT1* were obtained, namely NIL‐TT, NIL‐TA, and NIL‐AA.

### Dual Luciferase Reporter Assay

Based on the method described by Zhang,^[^ ^51^ ^]^ a plasmid was constructed for the functional analysis of the 3’UTR. The 3’UTRs of both genotypes, along with their corresponding 3‐kb promoters, were amplified using the genomic DNA of TS‐9 and TS‐222 as templates. Subsequently, the sequences of the 3’UTRs were integrated downstream of the *luc* sequence, while the 3‐kb promoters were integrated upstream to generate the reporter vector. The full‐length pre‐miRNA for *miR8762* was amplified from TS‐9 genomic DNA and then cloned into pHELLSGATE8 downstream of the CaMV35S promoter to generate the overexpression construct *35Spro: miR8762*. *Agrobacterium* strains containing a reporter construct and an effector construct were infiltrated into tobacco leaves. After 3 d of incubation, firefly luciferase (LUC) and *Renilla* luciferase (REN) activities were determined, and the ratios of LUC to REN were calculated to indicate transactivation activity. The primers used for the constructs are listed in Table S7 (Supporting Information).

### Detection of Domestication and Improvement Sweeps

To identify the selection region around *LCT1*, SNPs near *LCT1* in the tomato genome were obtained (corresponding to ch03: 64.0–67.0 Mb, SL2.50). VCFtools was used to measure the level of nucleotide diversity (π) and population differentiation statistic (*F*_ST_) using a 100‐kb window with a step size of 10 kb in PIM, CER, and BIG accessions. To identify selection regions during domestication, the ratio of π (π_wild_/π_CER_) and *F*_ST_ between wild and CER accessions were computed. Similarly, to identify selection regions during improvement, the ratio of π (π_CER_/π_BIG_) and *F*_ST_ between CER and BIG accessions were calculated. Thresholds for selection were set at the top 5% of π ratios and *F*_ST_. Domestication: π_wild_/π_CER_ ≥ 4.8053, *F*_ST_ (wild and CER) ≥ 0.6830. Improvement: π_CER_/π_BIG_ ≥ 9.0313, *F*_ST_ (CER and BIG) ≥ 0.1634.

### Subcellular Localization

The full‐length coding sequence of *LCT1* without the stop codon was amplified from cDNA prepared from TS‐9 and then cloned into 101LYFP.^[^ ^52^ ^]^ The CaMV35S: *LCT1*‐YFP construct was generated using homologous recombination. The fusion proteins of CaMV35S: *LCT1*‐YFP and the endoplasmic reticulum marker RFP‐HDEL were co‐injected and transiently expressed in the leaves of *Nicotiana benthamiana*.^[^ ^53^ ^]^ Fluorescence of the *N. benthamiana* leaf cells was examined and imaged using a confocal laser scanning microscope (SP8, LEICA) two days after infiltration.

### GUS Staining

To construct a *LCT1pro*: *GUS* transgene, the promoter of *LCT1*was amplified and cloned into an expression vector (pHELLSGATE8‐GUS) derived from pHELLSGATE8 that lacked the CaMV35S promoter. Histochemical staining was performed using at least three independent *LCT1pro*: *GUS* transgenic T_1_ lines as described previously.^[^ ^54^ ^]^

### Determination of Cd^2+^ Influx

NMT was employed to measure Cd^2+^ absorption. Tomato seedlings at 21 d post‐germination were selected, and their root surfaces were rinsed with ddH_2_O to remove the substrate. The seedlings were then transferred to 1/2 Hoagland nutrient solution containing 10 µm CdCl_2_ for 5 d before measuring Cd^2+^ absorption in roots and petioles. Three biological replicates were used for each material, with measurements taken from mature lateral root zones in roots and vascular bundle regions in petioles. The relevant solution compositions used in the experiment were as follows: 10 µm Cd^2+^ testing solution (10 µm CdCl_2_, 0.2 mm MES, pH 5.8); 50 µm Cd^2+^ calibration solution (50 µm CdCl_2_, 0.2 mm MES, pH 5.8); 5 µm Cd^2+^ calibration solution (5 µm CdCl_2_, 0.2 mm MES, pH 5.8); Cd^2+^ sensor backfill solution (10 mm Cd(NO_3_)_2_, 0.1 mm KCl).

### Statistical Analyses

All statistical analyses were performed using GraphPad Prism version 8.0 (http://www.graphpad.com/). One‐way ANOVA with Tukey's post hoc test and Student's *t*‐test were used in this study. Different letters above the bars indicate statistically significant differences (*p* < 0.05).

## Conflict of Interest

The authors declare no conflict of interest.

## Author Contributions

X.Z., M.Q., W.W., Z.Y., and Y.Z. conceptualized the study. Methodology was developed by X.Z., M.Q., J.T., F.L., P.G., and Y.Y. Investigation was carried out by X.Z., M.Q., and Y.L., with visualization by X.Z., and H.X. Funding acquisition was handled by Y.Z., while project administration was managed by Z.Y., and Y.Z. Supervision was provided by Y.Z. The original draft was written by X.Z., and review and editing were done by X.Z., D.G., Z.Y., and Y.Z.

## Supporting information



Supporting Information

Supporting Information

Supporting Information

Supporting Information

Supporting Information

Supporting Information

## Data Availability

Research data are not shared.

## References

[1] X. Liang , S. L. Yang , Z. C. Lou , A. Ali , Sustainability 2024, 16, 1285.

[2] H. Gui , Q. Yang , X. Lu , H. Wang , Q. Gu , J. D. Martín , Environ. Res. 2023, 222, 115328. 36693463 10.1016/j.envres.2023.115328

[3] Y. S. Xu , S. H. Xiang , X. Y. Zhang , H. J. Zhou , H. M. Zhang , J. Hazard. Mater. 2022, 439, 129575. 35863230 10.1016/j.jhazmat.2022.129575

[4] S. Soni , A. B. Jha , R. S. Dubey , P. Sharma , Sci. Total Environ. 2024, 912, 168826. 38042185 10.1016/j.scitotenv.2023.168826

[5] F. U. Haider , C. Liqun , J. A. Coulter , S. A. Cheema , J. Wu , R. Zhang , M. Wenjun , M. Farooq , Ecotoxicol. Environ. Saf. 2021, 211, 111887. 33450535 10.1016/j.ecoenv.2020.111887

[6] B. A. Gong , W. J. Nie , Y. Y. Yan , Z. X. Gao , Q. H. Shi , J. Hazard. Mater. 2017, 336, 202. 28494308 10.1016/j.jhazmat.2017.04.058

[7] S. M. Gallego , L. B. Pena , R. A. Barcia , C. E. Azpilicueta , M. F. Iannone , E. P. Rosales , M. S. Zawoznik , M. D. Groppa , M. P. Benavides , Environ. Exp. Bot. 2012, 83, 33.

[8] I. Suhani , S. Sahab , V. Srivastava , R. P. Singh , Curr. Opin. Toxicol. 2021, 27, 1.

[9] M. Rusin , J. Domagalska , D. Rogala , M. Razzaghi , I. Szymala , Sci. Rep. 2021, 11, 11913. 34099845 10.1038/s41598-021-91554-z PMC8184968

[10] Z. N. Wang , Y. Sun , W. B. Yao , Q. Ba , H. Wang , Front. Immunol. 2021, 12, 695484. 34354707 10.3389/fimmu.2021.695484 PMC8330548

[11] T. Inaba , E. Kobayashi , Y. Suwazono , M. Uetani , M. Oishi , H. Nakagawa , K. Nogawa , Toxicol. Lett. 2005, 159, 192. 16006079 10.1016/j.toxlet.2005.05.011

[12] M. Peana , A. Pelucelli , C. T. Chasapis , S. P. Perlepes , V. Bekiari , S. Medici , M. A. Zoroddu , Biomolecules 2023, 13, 36. 10.3390/biom13010036 PMC9855641 36671421

[13] E. Y. Hernandez‐Cruz , I. Amador‐Martinez , A. K. Aranda‐Rivera , A. Cruz‐Gregorio , J. P. Chaverri , Chem. Biol. Interact. 2022, 361, 109961. 35500868 10.1016/j.cbi.2022.109961

[14] S. Fu , Y. Lu , X. Zhang , G. Yang , D. Chao , Z. Wang , M. Shi , J. Chen , D.‐Y. Chao , R. Li , J. F. Ma , J. Xia , J. Exp. Bot. 2019, 70, 5909. 31328224 10.1093/jxb/erz335 PMC6812702

[15] A. Sasaki , N. Yamaji , K. Yokosho , J. F. Ma , Plant. Cell. 2012, 24, 2155. 22589467 10.1105/tpc.112.096925 PMC3442593

[16] H. Jia , X. Wang , T. Wei , R. Zhou , H. Muhammad , L. Hua , X. Ren , J. Guo , Y. Ding , Environ. Exp. Bot. 2019, 167, 103829.

[17] Q.‐Y. Lv , M.‐L. Han , Y.‐Q. Gao , C.‐Y. Zhang , Y.‐L Wang , Z.‐F. Chao , L.‐Y Zhong , D.‐Y. Chao , New Phytol. 2022, 235, 1486. 35510797 10.1111/nph.18201

[18] Y. Chen , Z.‐F. Chao , M. Jin , Y.‐L Wang , Y. Li , J.‐C. Wu , Y. Xiao , Y. Peng , Q.‐Y. Lv , S. Gui , X. Wang , M.‐L. Han , A. R. Fernie , D.‐Y. Chao , J. Yan , J. Genet. Genomics 2023, 50, 130. 36028132 10.1016/j.jgg.2022.08.003

[19] R. Ravichandran , M. Rajendran , D. Devapiriam , Food Chem. 2014, 146, 472. 24176370 10.1016/j.foodchem.2013.09.080

[20] R. Ren , Z. Cao , X. Ma , Z. Li , K. Zhao , Di Cao , Q. Ma , M. Hou , K. Zhao , L. Zhang , D. Qiu , F. Gong , X. Zhang , H. Liu , D. Yin , J. Pineal Res. 2025, 77, 70035. 10.1111/jpi.70035 PMC11822082 39940063

[21] Y. Lu , T. Li , R. Li , P. Zhang , X. Li , Z. Bai , J. Wu , J. Hazard. Mater. 2024, 479, 135655. 39217923 10.1016/j.jhazmat.2024.135655

[22] M. C. Begum , M. Islam , M. R. Sarkar , M. A. S Azad , A. K. M. N Huda , A. H. Kabir , J. Plant Interact. 2016, 11, 124.

[23] H. Etesami , Ecotoxicol. Environ. Saf. 2018, 147, 175. 28843189 10.1016/j.ecoenv.2017.08.032

[24] R. C. Liu , L. Yang , Y. N. Zou , Q. S. Wu , Hortic. Plant J. 2023, 9, 463.

[25] X. Cao , R. Du , Y. Xu , Y. Wu , K. Ye , J. Ma , Y. Lyu , T. Sun , X. Zhu , Z. Liu , J. Yin , G. Zhu , Z. Huang , H. Lyu , S. Huang , J. Zhang , Hortic. Plant J. 2024, 10, 1383.

[26] H. Hassan , S. H. Elaksher , S. Shabala , B. Ouyang , Plant Physiol. Biochem. 2024, 214, 108968. 39074436 10.1016/j.plaphy.2024.108968

[27] G. Zhu , S. Wang , Z. Huang , S. Zhang , Q. Liao , C. Zhang , T. Lin , M. Qin , M. Peng , C. Yang , X. Cao , X. Han , X. Wang , E. van der Knaap , Z. Zhang , X. Cui , H. Klee , A. R. Fernie , J. Luo , S. Huang , Cell 2018, 172, 249. 29328914 10.1016/j.cell.2017.12.019

[28] J. Ye , X. Wang , W. Wang , H. Yu , G. Ai , C. Li , P. Sun , X. Wang , H. Li , B. Ouyang , J. Zhang , Y. Zhang , H. Han , J. J. Giovannoni , Z. Fei , Z. Ye , Plant Physiol. 2021, 186, 2078. 34618111 10.1093/plphys/kiab230 PMC8331143

[29] D. Tieman , G. Zhu , M. F. R. Resende , T. Lin , C. Nguyen , D. Bies , J. L. Rambla , K. S. O. Beltran , M. Taylor , B. Zhang , H. Ikeda , Z. Liu , J. Fisher , I. Zemach , A. Monforte , D. Zamir , A. Granell , M. Kirst , S. Huang , H. Klee , Science 2017, 355, 391. 28126817 10.1126/science.aal1556

[30] Z. Wang , Y. Hong , G. Zhu , Y. Li , Q. Niu , J. Yao , K. Hua , J. Bai , Y. Zhu , H. Shi , S. Huang , J.‐K. Zhu , Embo. J. 2020, 39, 103256. 10.15252/embj.2019103256 PMC7232006 32134151

[31] M. J. L. Morton , M. Awlia , N. Al‐Tamimi , S. Saade , Y. Pailles , S. Negrão , M. Tester , Plant. J. 2019, 97, 148. 30548719 10.1111/tpj.14189 PMC6850516

[32] H. Yan , W. Xu , J. Xie , Y. Gao , L. Wu , L. Sun , Lu Feng , X. Chen , T. Zhang , C. Dai , T. Li , X. Lin , Z. Zhang , X. Wang , F. Li , X. Zhu , J. Li , Z. Li , C. Chen , M. Ma , H. Zhang , Z. He , Nat. Commun. 2019, 10, 2562. 31189898 10.1038/s41467-019-10544-y PMC6561962

[33] M. Yang , K. Lu , F.‐J. Zhao , W. Xie , P. Ramakrishna , G. Wang , Q. Du , L. Liang , C. Sun , H. Zhao , Z. Zhang , Z. Liu , J. Tian , X.‐Y. Huang , W. Wang , H. Dong , J. Hu , L. Ming , Y. Xing , G. Wang , J. Xiao , D. E. Salt , X. Lian , Plant. Cell. 2018, 30, 2720. 30373760 10.1105/tpc.18.00375 PMC6305983

[34] D. Z. Alomari , A. M. Alqudah , K. Pillen , N. Von Wirén , M. S. Röder , J. Exp. Bot. 2021, 72, 6305. 34145452 10.1093/jxb/erab297 PMC8483787

[35] B. Harr , M. Kauer , C. Schlötterer , Proc. Natl. Acad. Sci. USA. 2002, 99, 12949. 12351680 10.1073/pnas.202336899 PMC130566

[36] J. Maynard‐Smith , J. Haigh , Genet. Res. 1974, 23, 23. 4407212

[37] T. Lin , G. Zhu , J. Zhang , X. Xu , Q. Yu , Z. Zheng , Z. Zhang , Y. Lun , S. Li , X. Wang , Z. Huang , J. Li , C. Zhang , T. Wang , Y. Zhang , A. Wang , Y. Zhang , K. Lin , C. Li , G. Xiong , Y. Xue , A. Mazzucato , M. Causse , Z. Fei , J. J. Giovannoni , R. T. Chetelat , D. Zamir , T. Städler , J. Li , Z. Ye , et al., Nat. Genet. 2014, 46, 1220. 25305757 10.1038/ng.3117

[38] M. Chakrabarti , N. Zhang , C. Sauvage , S. Muños , J. Blanca , J. Cañizares , M. J. Diez , R. Schneider , M. Mazourek , J. McClead , M. Causse , E. van der Knaap , Proc. Natl. Acad. Sci. USA. 2013, 110, 17125. 24082112 10.1073/pnas.1307313110 PMC3801035

[39] X. Ji , B. Dong , B. Shiran , M. J. Talbot , J. E. Edlington , T. Hughes , R. G. White , F. Gubler , R. Dolferus , Plant Physiol. 2011, 156, 647. 21502188 10.1104/pp.111.176164 PMC3177265

[40] C.‐F. Su , Y.‐C Wang , T.‐H. Hsieh , C.‐A. Lu , T.‐H. Tseng , S.‐M. Yu , Plant Physiol. 2010, 153, 145. 20130099 10.1104/pp.110.153015 PMC2862423

[41] H. Yu , T. Lin , X. Meng , H. Du , J. Zhang , G. Liu , M. Chen , Y. Jing , L. Kou , X. Li , Q. Gao , Y. Liang , X. Liu , Z. Fan , Y. Liang , Z. Cheng , M. Chen , Z. Tian , Y. Wang , C. Chu , J. Zuo , J. Wan , Q. Qian , B. Han , A. Zuccolo , R. A. Wing , C. Gao , C. Liang , J. Li , Cell 2021, 184, 1156. 33539781 10.1016/j.cell.2021.01.013

[42] J. Zeng , C. Zhou , Z. He , Y. Wang , L. Xu , G. Chen , W. Zhu , Y. Zhou , H. Kang , Int. J. Mol. Sci. 2023, 24, 1609. 36675124 10.3390/ijms24021609 PMC9863149

[43] L. Chen , L. Duan , M. Sun , Z. Yang , H. Li , K. Hu , H. Yang , L. Liu , Front. Plant Sci. 2023, 13, 1052569. 36684716 10.3389/fpls.2022.1052569 PMC9846265

[44] T. Song , Y. Shi , L. Shen , C. Cao , Y. Shen , W. Jing , Q. Tian , F. Lin , W. Li , W. Zhang , Proc. Natl. Acad. Sci. USA. 2021, 118, 2114347118. 10.1073/pnas.2114347118 PMC8685926 34876526

[45] J. Hesselink , D. J. Kopsky , J. Neurol. 2017, 264, 1617. 28083647 10.1007/s00415-017-8391-5

[46] O. Viswanath , I. Urits , M. R. Jones , J. M. Peck , J. Kochanski , M. Hasegawa , B. Anyama , A. D. Kaye , Curr. Pain Headache Rep. 2019, 23, 37. 31044330 10.1007/s11916-019-0774-0

[47] S. Uraguchi , S. Mori , M. Kuramata , A. Kawasaki , T. Arao , S. Ishikawa , J. Exp. Bot. 2009, 60, 2677. 19401409 10.1093/jxb/erp119 PMC2692013

[48] L. Yin , H. Zhang , Z. Tang , J. Xu , D. Yin , Z. Zhang , X. Yuan , M. Zhu , S. Zhao , X. Li , X. Liu , Genom. Prot. Bioinfo. 2021, 19, 619. 10.1016/j.gpb.2020.10.007 PMC9040015 33662620

[49] B. Ouyang , Y. H. Chen , H. X. Li , C. J. Qian , S. L. Huang , Z. B. Ye , J. Horticult. Sci. Biotechnol. 2005, 80, 517.

[50] X. R. Zhang , R. Henriques , S. S. Lin , Q. W. Niu , N. H. Chua , Nat. Protoc. 2006, 1, 641. 17406292 10.1038/nprot.2006.97

[51] S. Zhang , Z. Jiao , L. Liu , K. Wang , D. Zhong , S. Li , T. Zhao , X. Xu , X. Cui , Plant Physiol. 2018, 178, 1631. 30305372 10.1104/pp.18.01137 PMC6288745

[52] Y. Zhang , R. Ming , M. Khan , Y. Wang , B. Dahro , W. Xiao , C. Li , J.‐H. Liu , Plant Biotechnol. J. 2022, 20, 183. 34510677 10.1111/pbi.13705 PMC8710834

[53] J. Z. Zang , V. Kriechbaumer , P. W. Wang , J. Plant Physiol. 2021, 264, 153473. 34298331 10.1016/j.jplph.2021.153473

[54] L. Shang , J. Song , H. Yu , X. Wang , C. Yu , Y. Wang , F. Li , Y. Lu , T. Wang , B. Ouyang , J. Zhang , R. M. Larkin , Z. Ye , Y. Zhang , Plant. Cell. 2021, 33, 3293. 34338777 10.1093/plcell/koab201 PMC8505859

