# Supplementary material for: Co‐Selection of Low Cadmium Accumulation and High Yield During Tomato Improvement
Source: Adv Sci (Weinh). 2025 Jul 26;12(39):e05138. doi: 10.1002/advs.202505138 (PMC12533394; doi:10.1002/advs.202505138)
Supplement: Supplementary file 1 — Supporting Information [file ADVS-12-e05138-s004.docx]

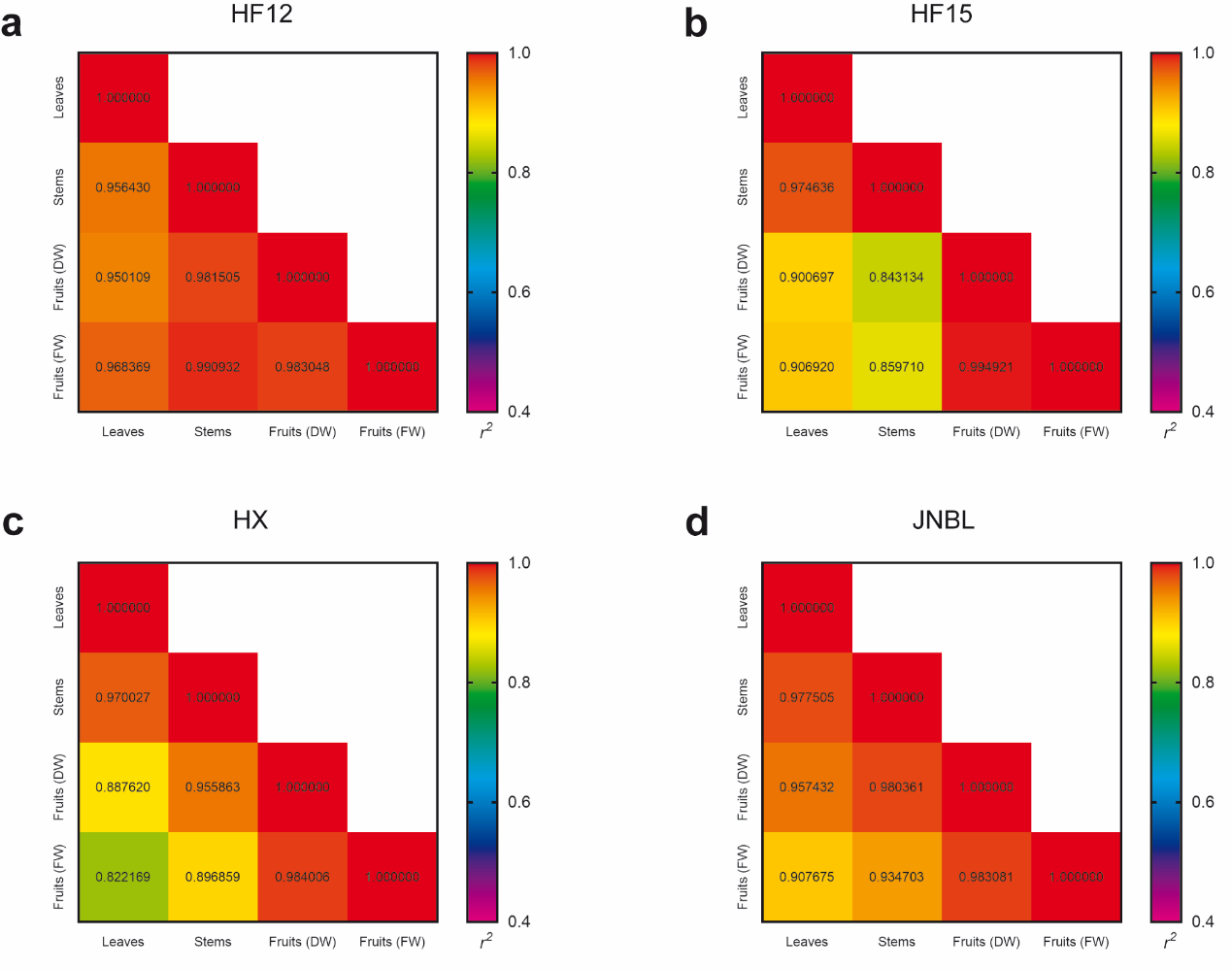


**Supplementary Fig. 1** Correlation analysis of Cd content between leaves, stems, fruits (DW), and fruits (FW). **a-d** The correlation coefficient for Cd content between leaves, stems, fruits (DW), and fruits (FW) of ‘HF12’ (**a**), ‘HF15’ (**b**), ‘HX’ (**c**), and ‘JNBL’ (**d**) cultivars. Values closer to 1 indicates a stronger correlation between two traits.


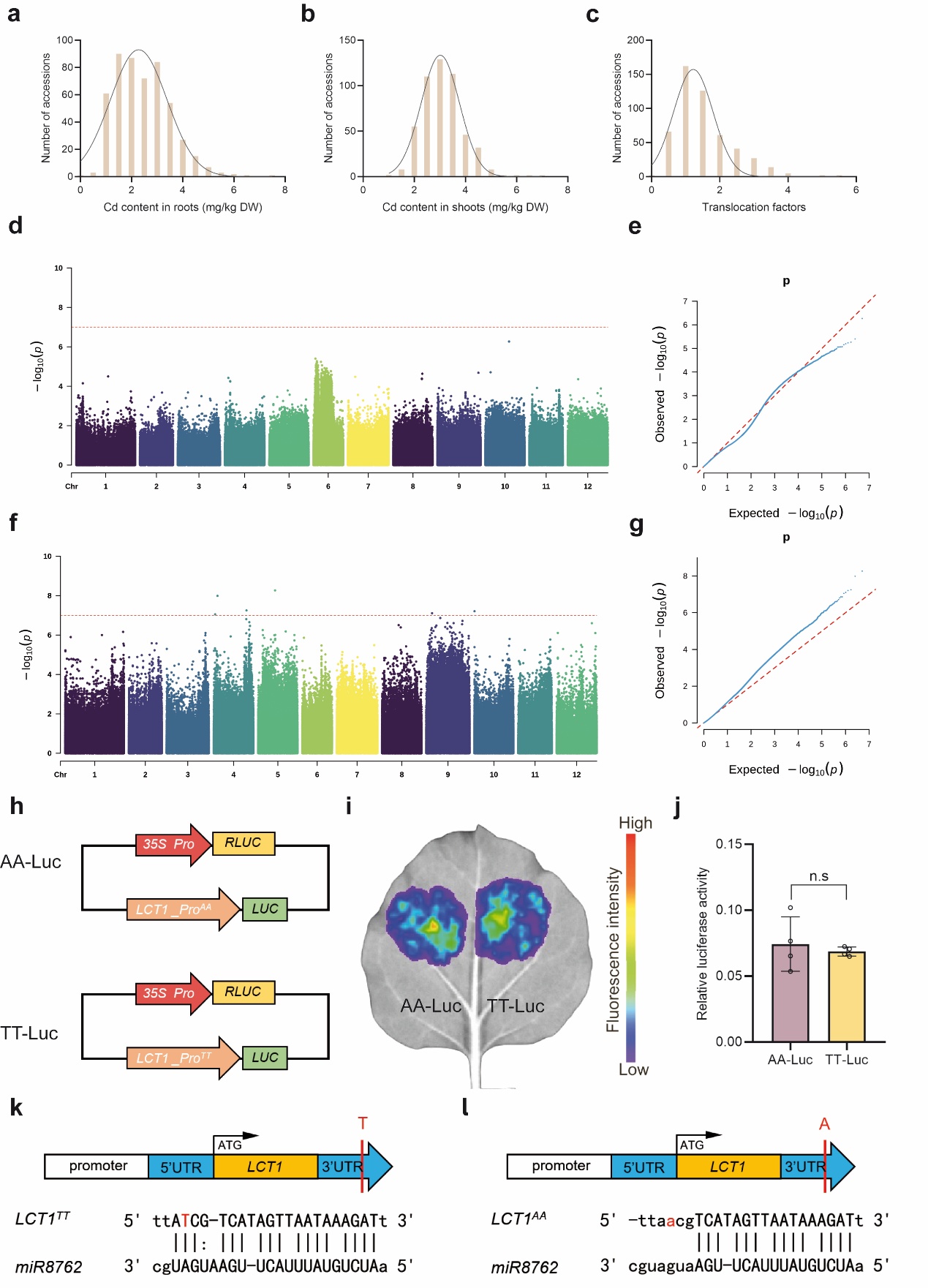


Supplementary Fig. 2 GWAS of root Cd content and translocation factor, the effect of promoter variation SNP15-T, and predicted function of SNP3-A. a-c Frequency distribution of Cd content in roots (a) and shoots (b), and frequency distribution of the translocation factor (c). Black curve indicates the fitted line of the distribution. d Manhattan plot of genome-wide association study for Cd content in roots. e Quantile–quantile plot for Cd content in roots. f Manhattan plot of genome-wide association study for translocation factor of Cd. g Quantile–quantile plot for translocation factor of Cd. The horizontal red lines represent the threshold for association analysis. h Schematic representation of the constructs used for the dual luciferase reporter assay. AA-Luc indicates that *Luc* expression is driven by the AA-type *LCT1* promoter, while TT-Luc indicates that *Luc* expression is driven by the TT-type *LCT1* promoter. i The effect of the two types of promoters on Luc fluorescence intensity. j The effect of the two types of promoters on Luc enzyme activity. *P*-value was determined by Student’s t-test, with *P* < 0.05 indicating significant difference. k The binding patterns between *miR8762* and *LCT1^TT^* predicted by an online tool (http://www.bioinformatics.com.cn). l The predicted binding patterns between *miR8762* and *LCT1^AA^*. Dashed lines between bases indicate partial complementarity, while solid lines indicate perfect complementarity. The red font highlights the key variant site SNP3-A in the 3’UTR of *LCT1*.


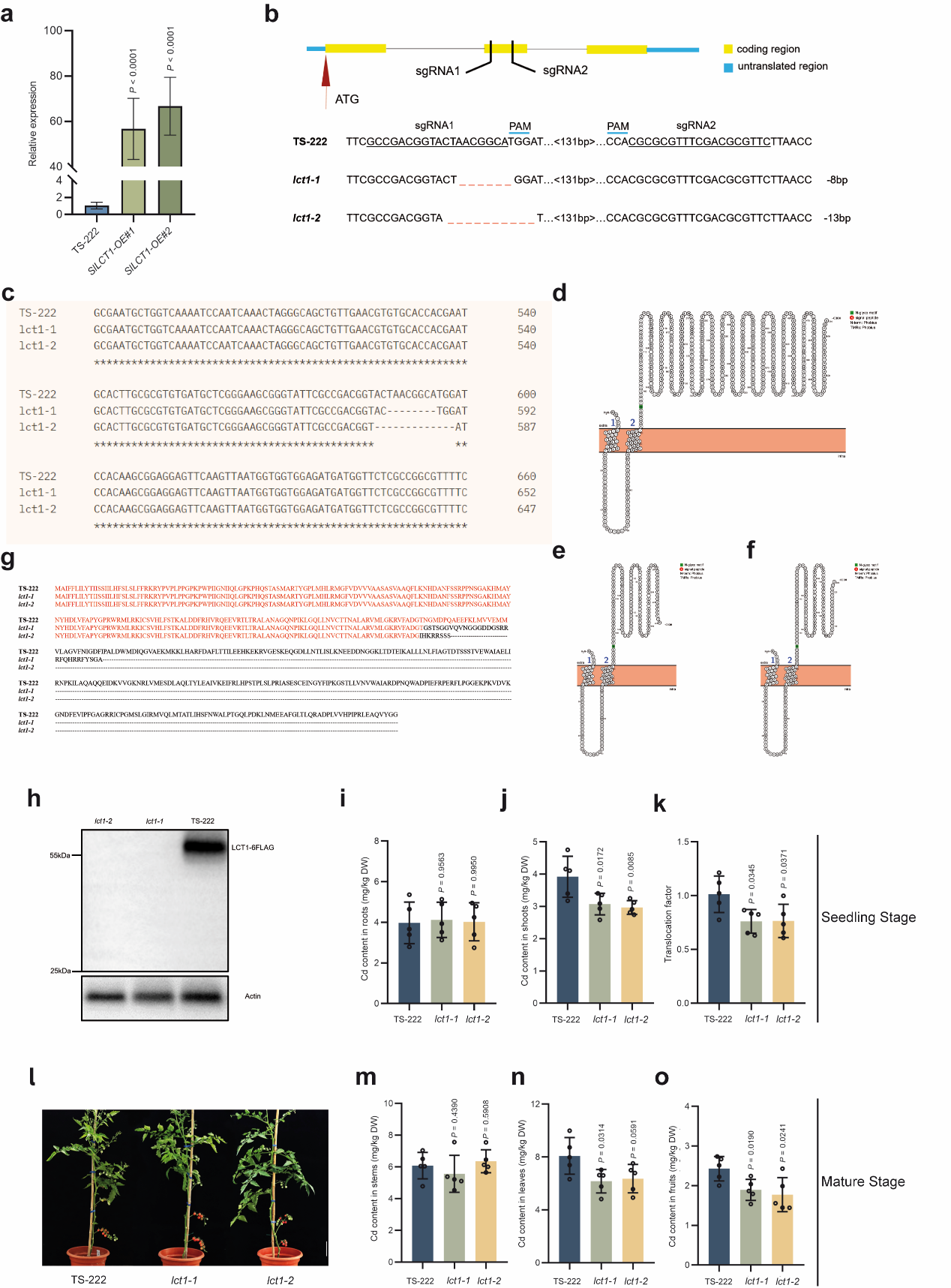


**Supplementary Fig. 3** Overexpression and CRISPR/Cas9-mediated knockout of *LCT1*. **a** Relative expression of *LCT1* in the overexpressing lines. **b** Mutations generated in *LCT1* by CRISPR/Cas9 using two independent single-guide RNA (sgRNA1 and sgRNA2). Sequences of the *LCT1* in wild-type (TS-222), and mutant tomato plants *lct1-1* and *lct1-2* are shown. The sgRNA-targeted sequences are underlined, and the protospacer adjacent motif (PAM) sequences are highlighted blue. Deletions are indicated by dashed lines. The red arrow indicates the position of the start codon. **c** cDNA sequence alignment between the *lct1* mutants and the wild-type TS-222. **d**-**f** The secondary structures of the LCT1 peptide chain in the wild-type TS-222 (**d**) and the *lct1-1* (**e**) *and lct1-2* (**f**) mutants. **g** Alignment of LCT1 amino acid sequence between *lct1* mutants and wild-type TS-222. **h** Detection of LCT1 protein after transient expression of wild-type TS-222, *lct1* and *lct1-2* mutants in tobacco. **i-k** Root Cd content (**i**), shoot Cd content (**j**), and Cd translocation factor (**k**) of wild type TS-222 and *lct1* mutants after 30 d of 1.0 mg/kg CdCl_2_ treatment during the seedling stage. **l** Growth of wild-type TS-222 (Same as Fig. 2j and Fig. 3j), *lct1-1* and *lct1-2* mutants under 1.0 mg/kg CdCl_2_ treatment. Scale bar, 12 cm. **m-o** Stems Cd content (**m**), leaves Cd content (**n**), and fruits Cd content (**o**) in wild-type TS-222, *lct1-1* and *lct1-2* mutants (The data for TS-222 are the same as those shown in Fig. 2k-m and Fig. 3l-n). *P-*values were determined by Student’s *t*-test, with *P* < 0.05 indicating significant difference.


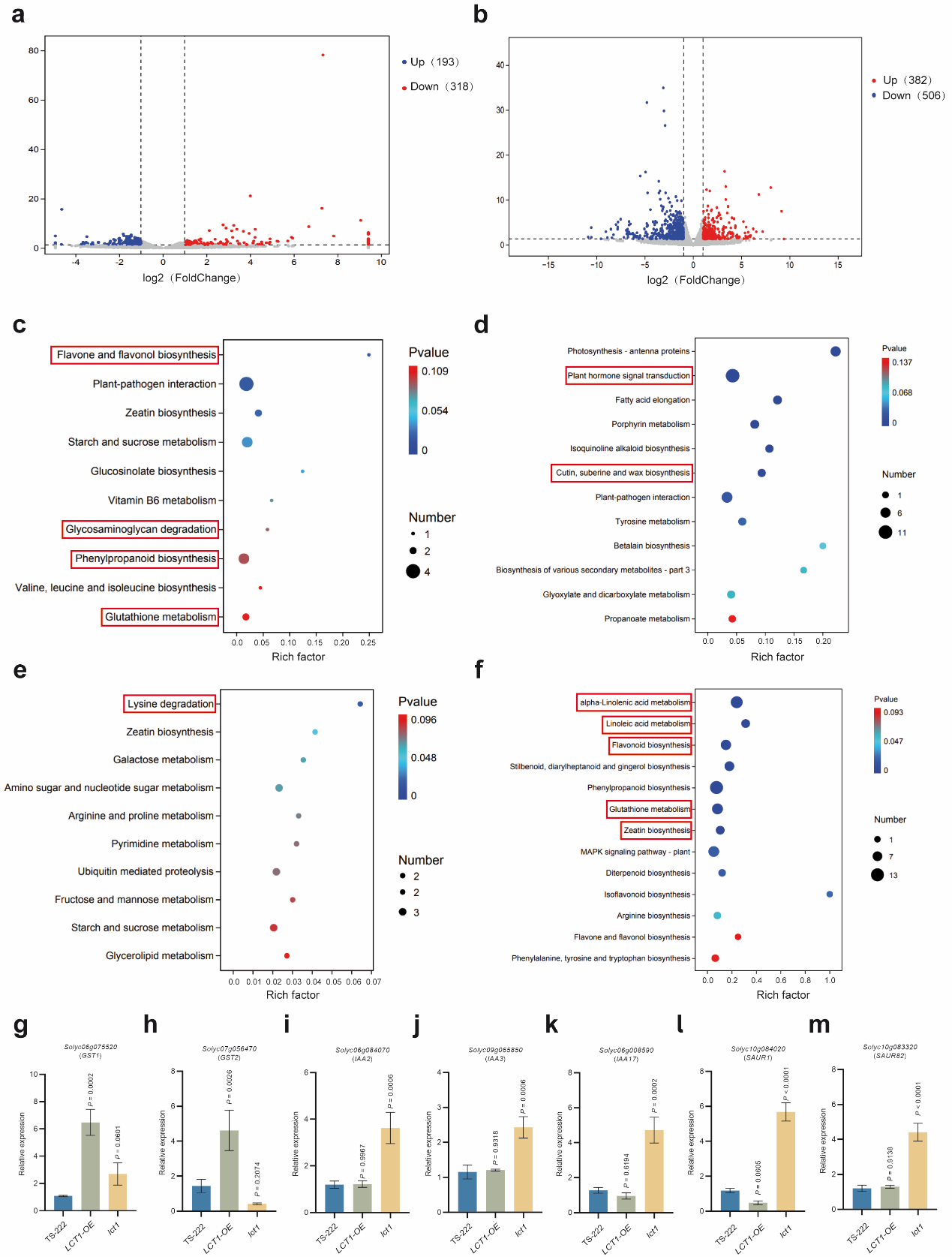


**Supplementary Fig. 4** RNA-seq analysis of *LCT1-OE* line and *lct1* mutant. **a** Volcano plot distribution of differentially expressed genes between the *LCT1-OE* line and wild-type TS-222. **b** Volcano plot distribution of differentially expressed genes between the *lct1* mutants and wild-type TS-222. **c-d** KEGG enrichment analysis of up-regulated genes in *LCT1-OE* line (**c**) and *lct1* mutants (**d**). **e-f** KEGG enrichment analysis of down-regulated genes in *LCT1-OE* line (**e**) and *lct1* mutants (**f**). Red boxes indicate pathways related to Cd accumulation. **g-h** Relative expression levels of up-regulated genes *GST1* (**g**) and *GST2* (**h**) in the *LCT1-OE* line. **i-m** Relative expression levels of up-regulated genes *IAA2* (**i**), *IAA3* (**j**), *IAA17* (**k**), *SAUR1* (**l**), and *SAUR82* (**m**) in the *lct1* mutants.


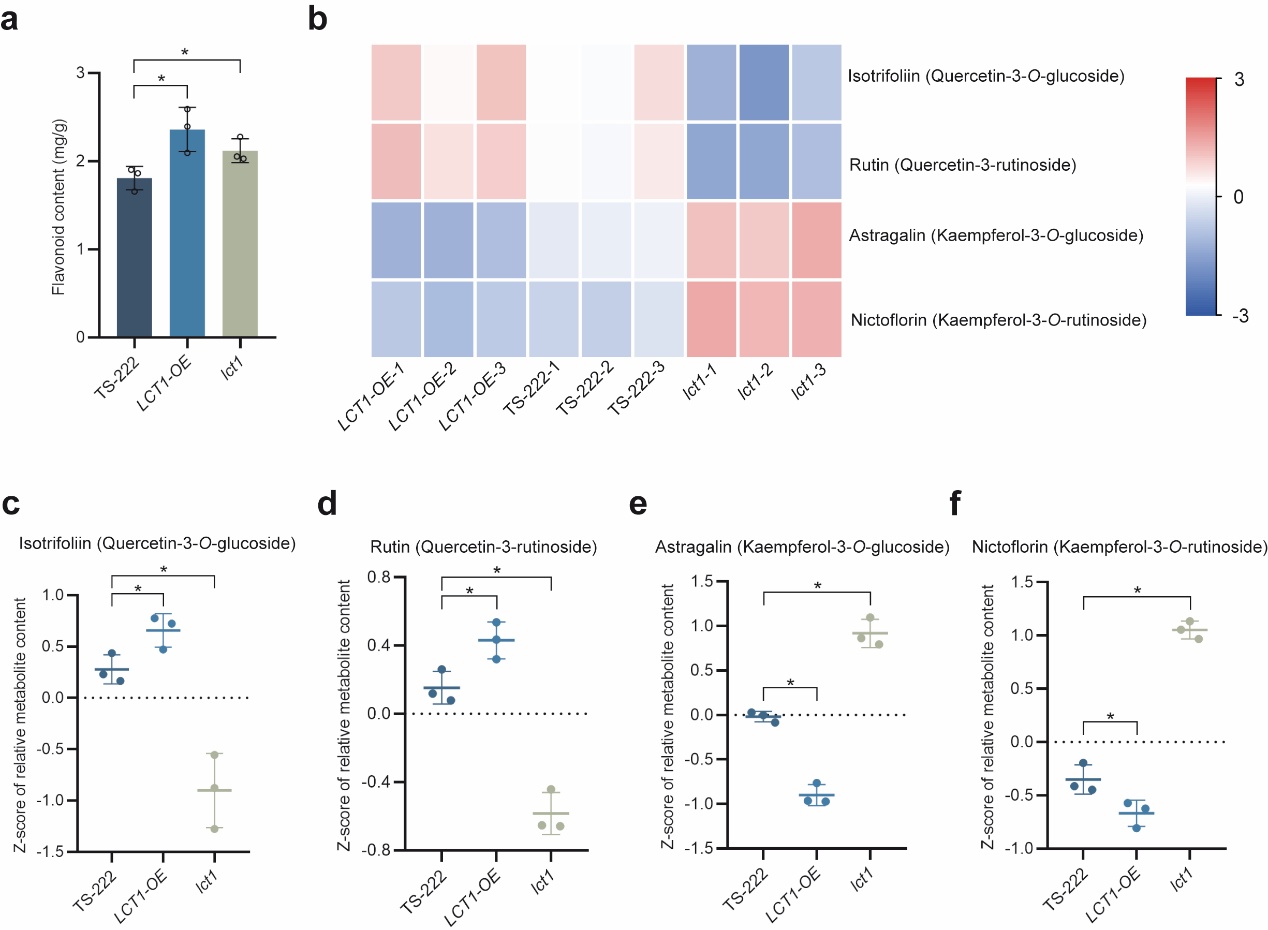


**Supplementary Fig. 5** Changes in the flavonoid contents in the *LCT1* overexpression line and the *lct1* mutant. **a** Total flavonoid content in wild-type TS-222, *LCT1-OE* line, and *lct1* mutant. **b** Heatmap illustrating variations in the relative content of four flavonoid metabolites. **c-f** The Z-score standardized contents of isotrifoliin (**c**), rutin (**d**), astragalin (**e**), and nictoflorin (**f**). An asterisk (*) denotes significant difference (*P* < 0.05).


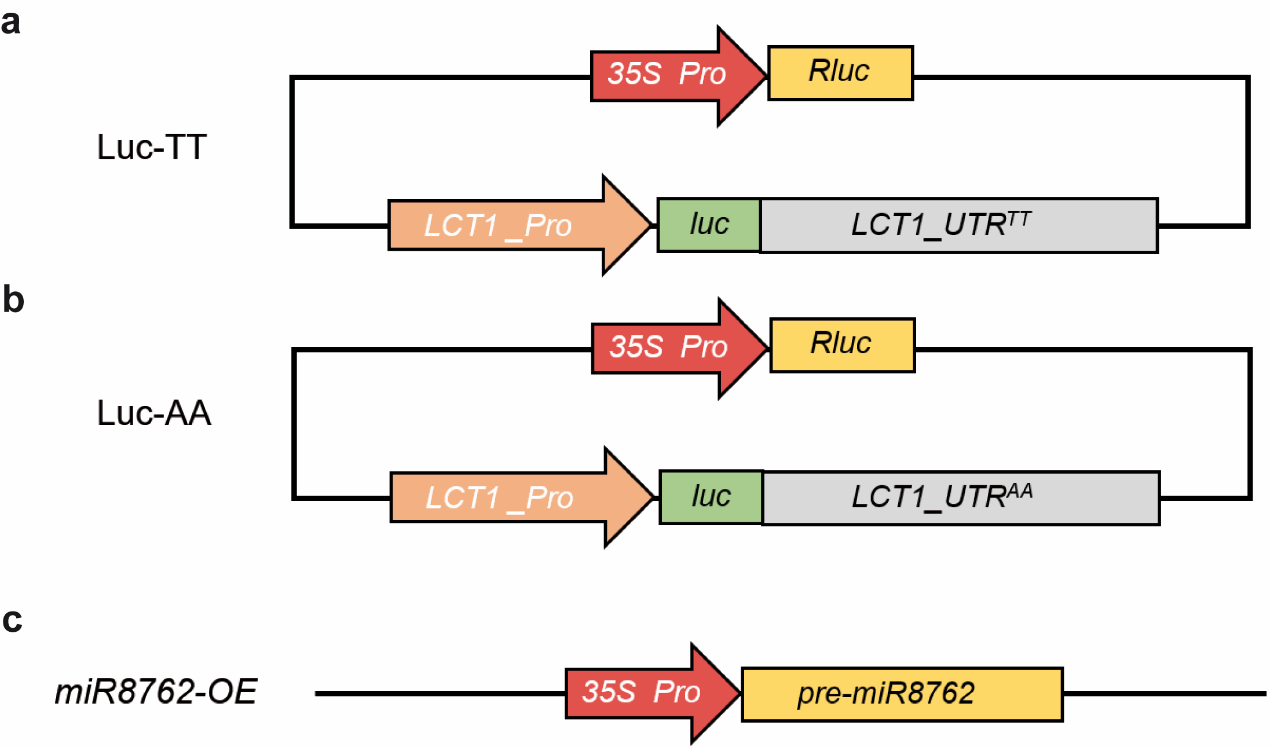


**Supplementary Fig. 6** Schematic representation of the constructs used for the dual luciferase reporter assay. **a** Vector construction for Luc-TT. The 3’ end of the *Luc* gene features the TT-type 3’UTR. **b** Vector construction for Luc-AA. The 3’ end of the *Luc* gene features the AA-type 3’UTR. Red and orange arrows represent the *35S* and *LCT1* promoters, respectively. Green and yellow boxes represent the coding sequences of firefly luciferase (Luc) and Renilla luciferase (Rluc), respectively. The gray box indicates the 3’UTR of the two *LCT1* genotypes. **c** Construction of the *miR8762* overexpressing vector *35S: pre-miR8762.* The red arrow indicates the *35S* promoter, and the yellow box represents the precursor sequence of *miR8762*.

**
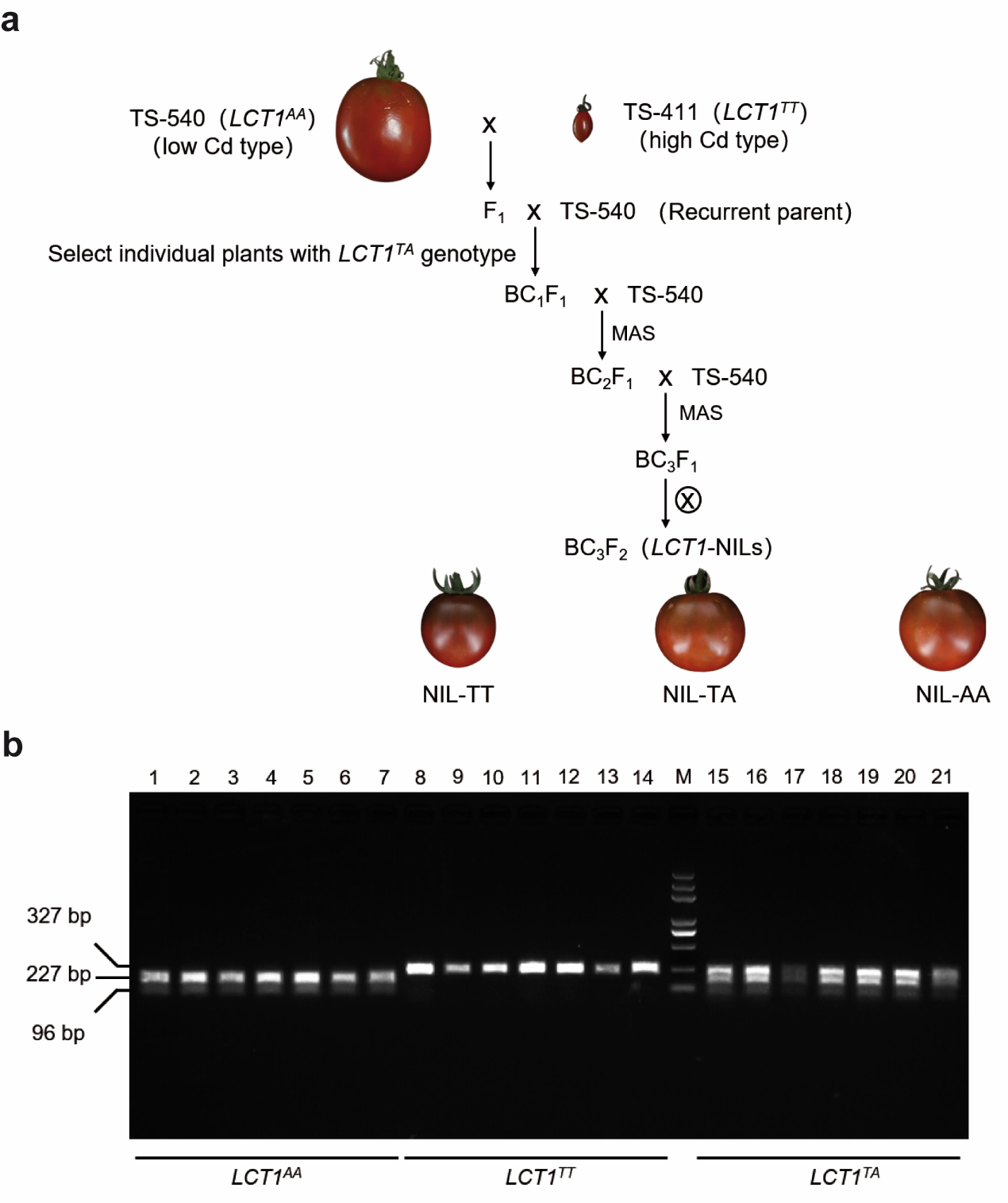
**

**Supplementary Fig. 7** Construction of *LCT1* near-isogenic lines. **a** The construction process for the NILs of *LCT1*. TS-540, a low Cd-accumulating extreme accession with the low Cd allele *LCT1^AA^*, and TS-411, a high Cd-accumulating extreme accession with the high Cd genotype *LCT1^TT^*, are used as the parental lines. TS-540 serves as the recurrent parent in successive backcrosses, during which LCT1-CAPS molecular markers designed based on the SNP variation in the 3’UTR of *LCT1* are used to select individuals containing the *LCT1^TA^* genotype. After three generations of backcrossing, one generation of selfing is performed to obtain the NILs of *LCT1*, namely NIL-TT, NIL-TA, and NIL-AA. **b** LCT1-CAPS molecular markers used in the construction of *LCT1* NILs. Lanes 1-7 represent individuals with the *LCT1^AA^* genotype, exhibiting a PCR band pattern of 227 bp + 96 bp. Lanes 8-14 represent individuals with the *LCT1^TT^* genotype, exhibiting a PCR band pattern of 327 bp. Lanes 15-21 represent individuals with the *LCT1^TA^* genotype, exhibiting a PCR band pattern of 327 bp + 227 bp + 96 bp.


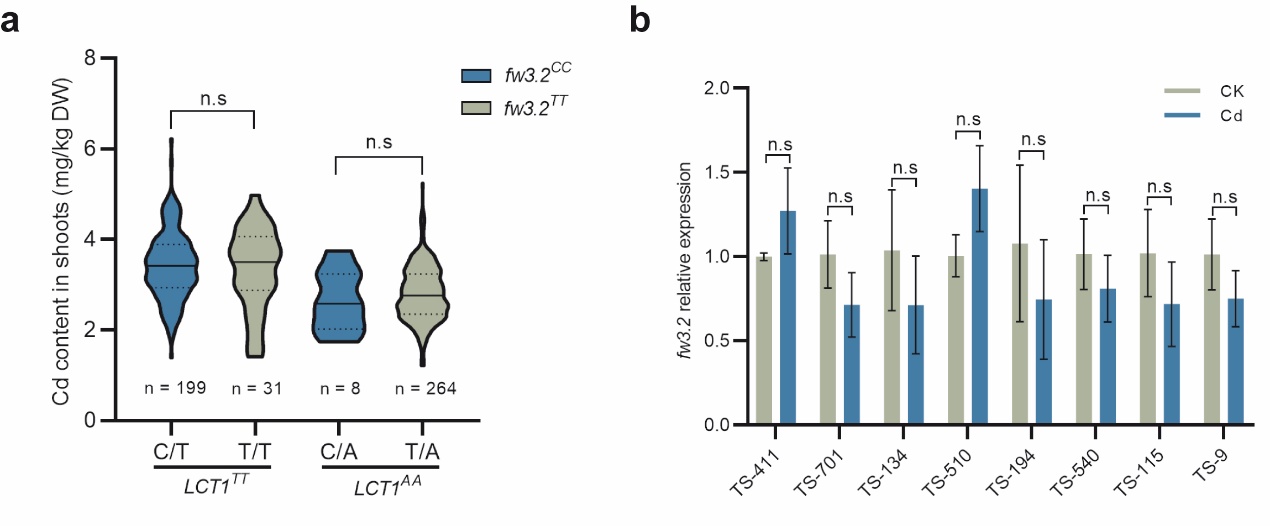


**Supplementary Fig. 8** *fw3.2* does not affect the Cd accumulation in tomato. **a** The Cd content in shoots corresponding to the four genotype combinations of *LCT1* and *fw3.2*. C/T denotes the accessions with *fw3.2^CC^* and *LCT1^TT^*. T/T denotes the accessions with *fw3.2^TT^* and *LCT1^TT^*. C/A denotes the accessions with *fw3.2^CC^* and *LCT1^AA^*. T/A denotes the accessions with *fw3.2^TT^* and *LCT1^AA^*. *n* indicates the number of accessions belonging to each combination. **b** Changes in *fw3.2* gene expression levels in high and low Cd-accumulating tomato accessions after 30 d of treatment with 0 mg/kg (CK) or 1.0 mg/kg CdCl_2_ (Cd). *P-*values were determined by Student’s *t*-test, with *P* < 0.05 indicating significant difference.


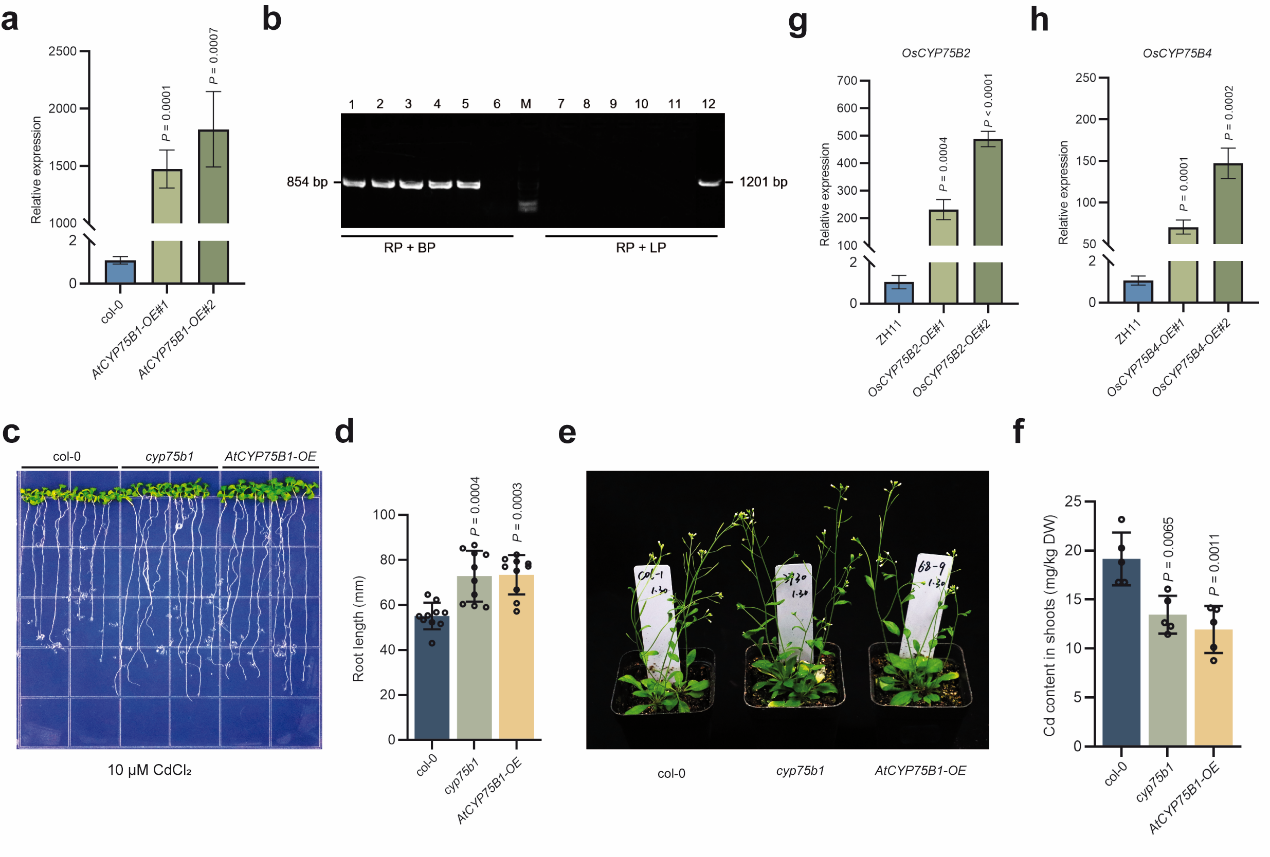


**Supplementary Fig. 9** Expression levels of *AtCYP75B1*, *OsCYP75B2*, and *OsCYP75B4*, and Cd accumulation in *Arabidopsis cyp75b1* mutants. **a** Relative expression levels of *AtCYP75B1* in the overexpression lines. **b** Positive detection of T-DNA insertion mutants *cyp75b1* for *Arabidopsis*. Lanes 1-5 and 7-11 are homozygous *cyp75b1* mutants, while lanes 6 and 12 are wild-type Col-0. LP and RP are primers flanking the T-DNA insertion site in the *Arabidopsis* genome. and BP is a primer on the T-DNA segment. **c-d** Root length of wild-type Col-0, mutant *cyp75b1*, and *AtCYP75B1-OE* line after 14 d of growth on 1/2 MS medium supplemented with 10 μM CdCl_2_. **e-f** Growth and shoot Cd content of potted wild-type Col-0, mutant *cyp75b1*, and *AtCYP75B1-OE* line after 30 d of 1.5 mg/kg CdCl_2_ treatment. **g-h** Relative expression levels of *OsCYP75B2* (g) and *OsCYP75B4* (h). *P-*values were determined by Student’s *t*-test, with *P* < 0.05 indicating significant difference.


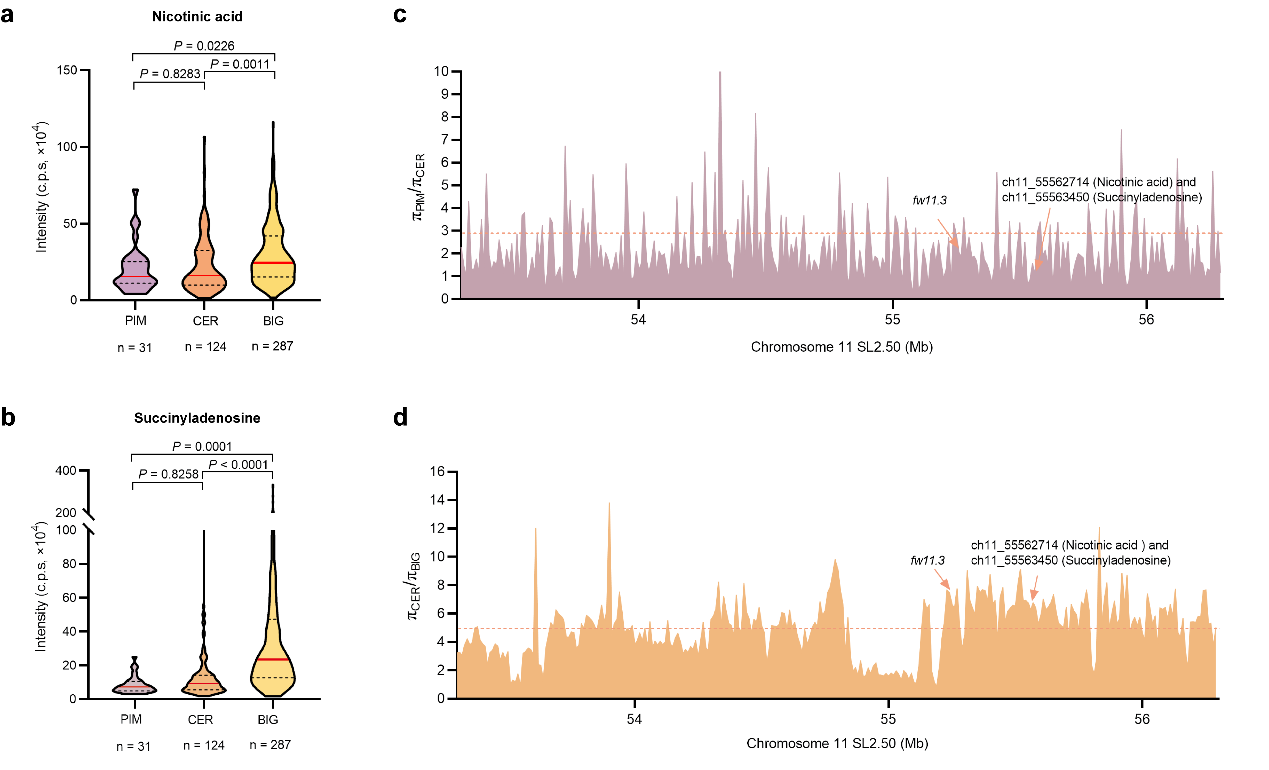


**Supplementary Fig. 10** Co-selection of nicotinic acid and succinyladenosine with fruit weight. **a-b** Relative contents of nicotinic acid (**a**) and succinyladenosine (**b**) in three groups of 442 tomato accessions. The red solid line represents the median, while the black dashed lines represent the first and third quartiles. **c-d** Ratios of nucleotide diversity (π) surrounding the *fw11.3* and GWAS-identified lead SNPs for nicotinic acid and succinyladenosine between PIM and CER accessions (**c**) and between CER and BIG (**d**) accessions on chromosome 11. The red horizontal dashed line indicates the top 5% threshold for the entire genome. The location of *fw11.3* and the lead SNPs for nicotinic acid and succinyladenosine on chromosome 11 is marked by the red arrow. Source data are from Zhu et al (2018).


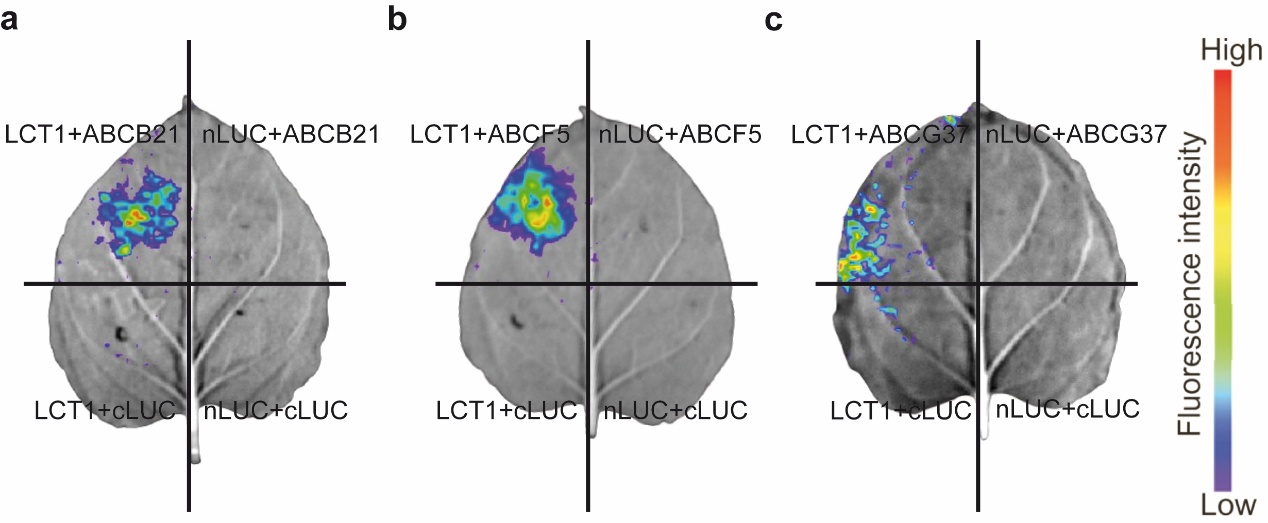


**Supplementary Fig. 11** Potential transporters interacting with LCT1. **a** Split luciferase complementation assay showing the interaction between LCT1 and ABCB21 (*Solyc03g114960*). **b** Split luciferase complementation assay showing the interaction between LCT1 and ABCF5 (*Solyc04g051800*). **c** Split luciferase complementation assay showing the interaction between LCT1 and ABCG37 (*Solyc01g101070*).

**Supplementary Tables**

**Supplementary Table1. 506 tomato accessions used in this study.**

See supplementary files.

**Supplementary Table2. The Cd content in roots and shoots, and translocation factors of 506 accessions.**

See supplementary files.

**Supplementary Table5.** **The geographic origin of the tomato accessions.**

See supplementary files.

**Supplementary Table7.** **List of primers used in this study.**

See supplementary files.

**Supplementary Table3. The candidate genes of *LCT1*.**

| **Lead SNPs** | ***P*** | **Location** | **Genes** | **SNP effect** | **Gene description** |
| --- | --- | --- | --- | --- | --- |
| 64869851 | 9.09E-11 | Promoter | Solyc03g115010.1 | none | Transcription factor, TCP |
| 64769236 | 9.93E-09 | Promoter | Solyc03g114890.2 | none | COBRA-like protein |
| 64837546 | 4.74E-09 | Promoter | Solyc03g114980.2 | none | Unknown Protein |
| 64922295 | 3.23E-08 | Promoter | Solyc03g115120.1 | none | Heat shock protein |
| 65007638 | 2.18E-08 | Promoter | Solyc03g115220.2 | none | Flavonoid 3’hydroxylase, F3’H |
| 65004108 | 2.51E-08 | Exon |  | synonymous |  |
| 65001793 | 3.48E-09 | 3'UTR |  | miR8762 |  |
| 65033600 | 4.35E-08 | Promoter | Solyc03g115240.2 | none | RNA polymerase-associated protein LEO1 |
| 65020764 | 5.20E-08 | Intron |  | none |  |
| 65033769 | 7.00E-08 | Promoter |  | none |  |
| 65034246 | 9.59E-08 | Promoter |  | none |  |
| 65034247 | 9.59E-08 | Promoter |  | none |  |
| 65052687 | 2.00E-08 | Intron | Solyc03g115250.2 | none | Genomic DNA chromosome 5 P1 clone MXM12 |
| 65054727 | 3.02E-08 | Intron |  | none |  |
| 65052631 | 3.67E-08 | Intron |  | none |  |
| 65069482 | 8.78E-08 | Intron | Solyc03g115290.2 | none | Unknown Protein |
| 64978528 | 2.54E-09 | Intergenic |  |  |  |
| 64766273 | 3.76E-09 | Intergenic |  |  |  |
| 64871576 | 7.46E-09 | Intergenic |  |  |  |
| 64978968 | 7.78E-09 | Intergenic |  |  |  |
| 65038615 | 8.17E-09 | Intergenic |  |  |  |
| 64899693 | 1.11E-08 | Intergenic |  |  |  |
| 64968796 | 1.18E-08 | Intergenic |  |  |  |
| 64862318 | 1.23E-08 | Intergenic |  |  |  |
| 64924410 | 2.10E-08 | Intergenic |  |  |  |
| 65038616 | 2.91E-08 | Intergenic |  |  |  |
| 65036339 | 4.19E-08 | Intergenic |  |  |  |

The gene denoted in red, *SlF3’H*, selected based on SNP effects and gene annotations, is designated for further functional studies.

**Supplementary Table4. The QTLs associated with fruit weight.**

| **Locus** | **gene ID** | **Year** | **Author** | **Article** |
| --- | --- | --- | --- | --- |
| *fw11.3* | Solyc11g071940 | 2017 | Mu, et al. | Fruit weight is controlled by *Cell Size Regulator* encoding a novel protein that is expressed in maturing tomato fruits |
| *fascinated* | Solyc11g071810 | 2008 | Cong, et al. | Regulatory change in YABBY-like transcription factor led to evolution of extreme fruit size during tomato domestication |
| *fw3.2* | Solyc03g114940 | 2013 | Chakrabart, et al. | A cytochrome P450 regulates a domestication trait in cultivated tomato |
| *lc* | Solyc02g083940 | 2011 | Munos, et al. | Increase in tomato locule number is controlled by  two single-nucleotide polymorphisms located near*WUSCHEL* |
| *fw2.2* | Solyc02g090730 | 2000 | Frary, et al. | *fw2.2*: a quantitative trait locus key to the evolution of tomato fruit size |
| *GBP1* | Solyc10g008950 | 2020 | Musseau, et al. | The tomato guanylate-binding protein SlGBP1  enables fruit tissue differentiation by maintaining  endopolyploid cells in a non-proliferative state |
| *CLV3* | Solyc11g071380 | 2015 | Xu, et al. | A cascade of arabinosyltransferases controls shoot meristem size in tomato |
| *ENO* | Solyc03g117230 | 2020 | Yuste-Lisbona, et al. | *ENO* regulates tomato fruit size through the floral meristem development network |
| *GRAS2*(*fw7.3*） | Solyc07g063940 | 2018 | Li, et al. | Silencing *GRAS2* reduces fruit weight in tomato |

**Supplementary Table 6. Potential interacting proteins of F3’H**

| **Gene ID** | **Annotation** |
| --- | --- |
| *Solyc08g082170* | Pectinlyase-like superfamily protein |
| *Solyc10g008340* | Structural constituent of cellwall |
| *Solyc02g065170* | L-ascorbateoxidaselike |
| ***Solyc03g114960*** | **ABC transporter B family protein** |
| *Solyc02g083500* | cel wall integrity/stress response component |
| *Solyc09g091840* | Glutathione Reductase |
| ***Solyc04g051800*** | **ABC transporter family protein** |
| *Solyc08g006790* | Earlynodulin-like protein |
| *Solyc06g009190* | Pectinesterase |
| *Solyc10g017850* | Peroxisomal membrane protein11-1 |
| *Solyc00g030000* | Cellulose synthase |
| *Solyc01g102350* | Pectin acetylesterase family protein |
| ***Solyc01g101070*** | **Pleiotropic drug resistance ABC transporter** |
| *Solyc12g043000* | Coatomer subunit beta-like protein |
| *Solyc06g072465* | 50S ribosomal protein L29 |
| *Solyc10g081370* | small nuclear ribonucleo protein F |
| *Solyc01g089970* | Nucleoside diphosphate kinase |
| *Solyc05g053650* | 26S proteasomenon-ATPase regulatory subunit 1 |
| *Solyc01g088020* | Transducin/WD40 repeat-like superfamily protein |
| *Solyc09g076040* | Protein Suppressor of Gene Silencing 3 |

The bold part is the ABC family transporter that interacts with F3’H in the IP-MS results.
